# SH003 suppresses breast cancer growth by accumulating p62 in autolysosomes

**DOI:** 10.18632/oncotarget.11393

**Published:** 2016-08-19

**Authors:** Youn Kyung Choi, Sung-Gook Cho, Yu-Jeong Choi, Yee Jin Yun, Kang Min Lee, Kangwook Lee, Hye-Hyun Yoo, Yong Cheol Shin, Seong-Gyu Ko

**Affiliations:** ^1^ Jeju International Marine Science Center for Research and Education, Korea Institute of Ocean Science and Technology (KIOST), Jeju, 695-975, Korea; ^2^ Department of Biotechnology, Korea National University of Transportation, Chungbuk, 368-701, Korea; ^3^ Department of Cancer Preventive Material Development, Graduate School, Kyung Hee University, Seoul, 130-701, Korea; ^4^ Department of Science in Korean Medicine, Graduate School, Kyung Hee University, Seoul, 130-701, Korea; ^5^ Institute of Pharmaceutical Science and Technology and Collage of Pharmacy, Hanyang University, Gyonggi, 426-791, Korea; ^6^ Laboratory of Clinical Biology and Pharmacogenomics, Department of Preventive Medicine, College of Korean Medicine, Kyung Hee University, Seoul, 130-701, Korea

**Keywords:** SH003, breast cancer, autophagy, apoptosis, p62

## Abstract

Drug markets revisits herbal medicines, as historical usages address their therapeutic efficacies with less adverse effects. Moreover, herbal medicines save both cost and time in development. SH003, a modified version of traditional herbal medicine extracted from *Astragalus membranaceus* (Am), *Angelica gigas* (Ag), and *Trichosanthes Kirilowii Maximowicz* (Tk) with 1:1:1 ratio (w/w) has been revealed to inhibit tumor growth and metastasis on highly metastatic breast cancer cells, both *in vivo* and *in vitro* with no toxicity. Meanwhile, autophagy is imperative for maintenance cellular homeostasis, thereby playing critical roles in cancer progression. Inhibition of autophagy by pharmacological agents induces apoptotic cell death in cancer cells, resulting in cancer treatment. In this study, we demonstrate that SH003-induced autophagy via inhibiting STAT3 and mTOR results in an induction of lysosomal p62/SQSTM1 accumulation-mediated reactive oxygen species (ROS) generation and attenuates tumor growth. SH003 induced autophagosome and autolysosome formation by inhibiting activation of STAT3- and mTOR-mediated signaling pathways. However, SH003 blocked autophagy-mediated p62/SQSTM1 degradation through reducing of lysosomal proteases, Cathepsins, resulting in accumulation of p62/SQSTM1 in the lysosome. The accumulation of p62/SQSTM1 caused the increase of ROS, which resulted in the induction of apoptotic cell death. Therefore, we conclude that SH003 suppresses breast cancer growth by inducing autophagy. In addition, SH003-induced p62/SQSTM1 could function as an important mediator for ROS generation-dependent cell death suggesting that SH003 may be useful for treating breast cancer.

## INTRODUCTION

Balance between protein synthesis and degradation is important to maintain cellular homeostasis. Protein degradation in eukaryotic cells follows one of systems, either proteasomal or lysosomal degradation system [1, 2]. Autophagy requires complex molecular mechanisms. Autophagic vesicle engulfs protein aggregates, damaged organelles, bacteria and other molecules, fusing with lysosome to form autophagosome. This autophagic mechanism degrades macromolecules by lysosomal hydrolytic enzymes including glucuronidase, ribonuclease, acid phosphatase, sulfatase and collagenase, and recycles constituent amino acids [3, 4]. So, when cells lack nutrients, autophagy is induced to supply nutrient for cell growth, metabolism and survival [2]. Alterations in autophagy occur in various diseases including vascular instability [5], metabolic dysfunction [6], cardiomyopathies and myopathies [7, 8], neurodegeneration [9], non-alcoholic fatty liver disease [10] and Crohn’s disease [11]. In addition, autophagy has an critical role in cancer [12]. While autophagy prevents tumorigenesis by inhibiting an accumulation of damaged-organelles and misfolded-proteins in normal cells, it activates cancer development by increasing tumor cell survival mechanism [13]. Moreover, autophagy regulates distant metastases of cancer cells [14, 15]. Therefore, targeting autophagy is important in cancer treatment [16–20].

Therefore, we recently developed a new herbal medicine named SH003 on the basis of the theory of the traditional Chinese medicine, and reported that it has an anti-cancer effect [21]. SH003 consists *Astragalus membranaceus* (Am), *Angelica gigas* (Ag), and *Trichosanthes Kirilowii* Maximowicz (Tk). Several studies showed that each herb has anti-cancer effects in different cancer cell types such as myeloid tumor [22], colon cancer [23], prostate cancer [24], liver cancer [25], non-small cell lung cancer [26] and breast cancer [27]. Nevertheless, it is yet unclear whether each herbal component or SH003 prevents cancer growth via autophagy. Here, we found that SH003 induced autophagy-mediated apoptosis through p62 accumulation-mediated ROS generation, thereby suggesting that SH003 may be useful for treating cancer.

## RESULTS

### SH003 induces apoptosis of breast cancer cells

**Figure 1:**
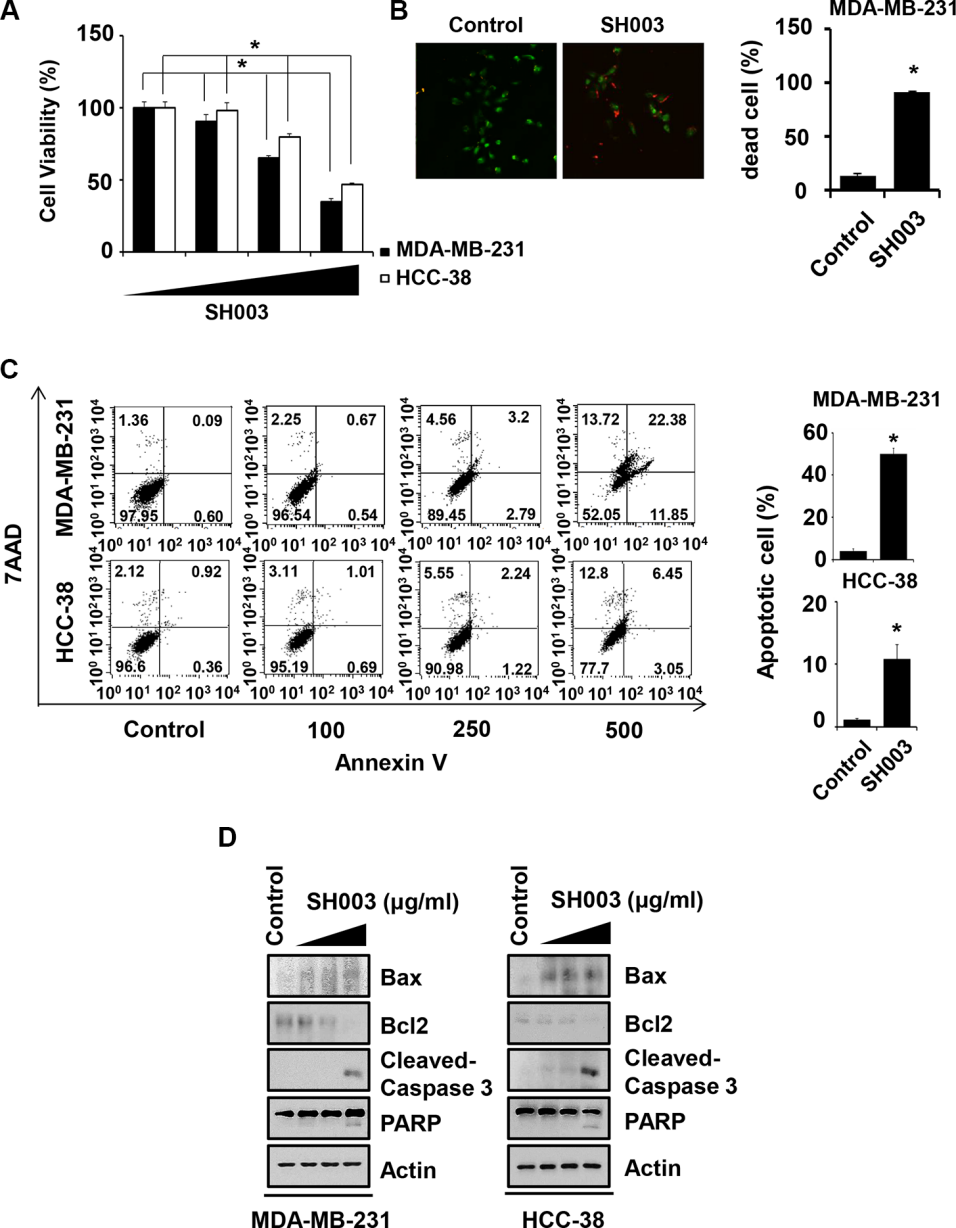
SH003 induces apoptotic cell death (**A**) Cell viability was measured by MTT assay. MDA-MB-231 and HCC-38 cells were seed in 96-well plates and treated with various concentration of SH003 (0, 100, 250 and 500 μg/ml) for 48 hours. Data were analyzed by ANOVA with *P* < 0.05. (**B**) After MDA-MB-231 cells were treated with 500 μg/ml of SH003 for 48 hours, live and dead assay was done by using live and dead cell assay kit. Dead cells (Red fluorescence-positive cells) were counted. *P < 0.05. (**C**) MDA-MB-231 and HCC-38 cells were treated with different doses of SH003 for 48 hours. Cells were stained with Annexin V and 7AAD at room temperature in the dark. Annexin V-positive apoptotic cells were detected using FACSCalibur. *P < 0.05. Graph shows annexin V-positive apoptotic cells (%) calculated from the total amount of right-upper and -lower portion. (**D**) Cells were treated with 500 μg/ml of SH003 for 24 hours and then performed western blots with anti-Bax, -Bcl2, -Cleaved caspase 3 and PARP. Actin was used for the internal control. Experiments were performed in triplicate. Bars indicate means that standard deviations (SD).

Breast cancer cells were treated with different concentrations (0, 100, 250 and 500 μg/ml) of SH003 for 48 hours and then subjected to cell viability assays. SH003 inhibited both MDA-MB-231 and HCC-38 breast cancer cell viabilities (Figure 1A). Our live and dead assays confirmed that SH003 increased dead cell numbers (Figure 1B). Furthermore, SH003 increased Annexin V-positive apoptotic cell numbers in a dose-dependent manner (Figure 1C). SH003 also altered Bax/Bcl2 ratio and induced cleavages of Caspase-3 and PARP (Figure 1D). Therefore, our data confirmed that SH003 induces apoptosis of breast cancer cells, consistently with our previous study [21].

### SH003 induces autophagy by inhibiting STAT3

**Figure 2:**
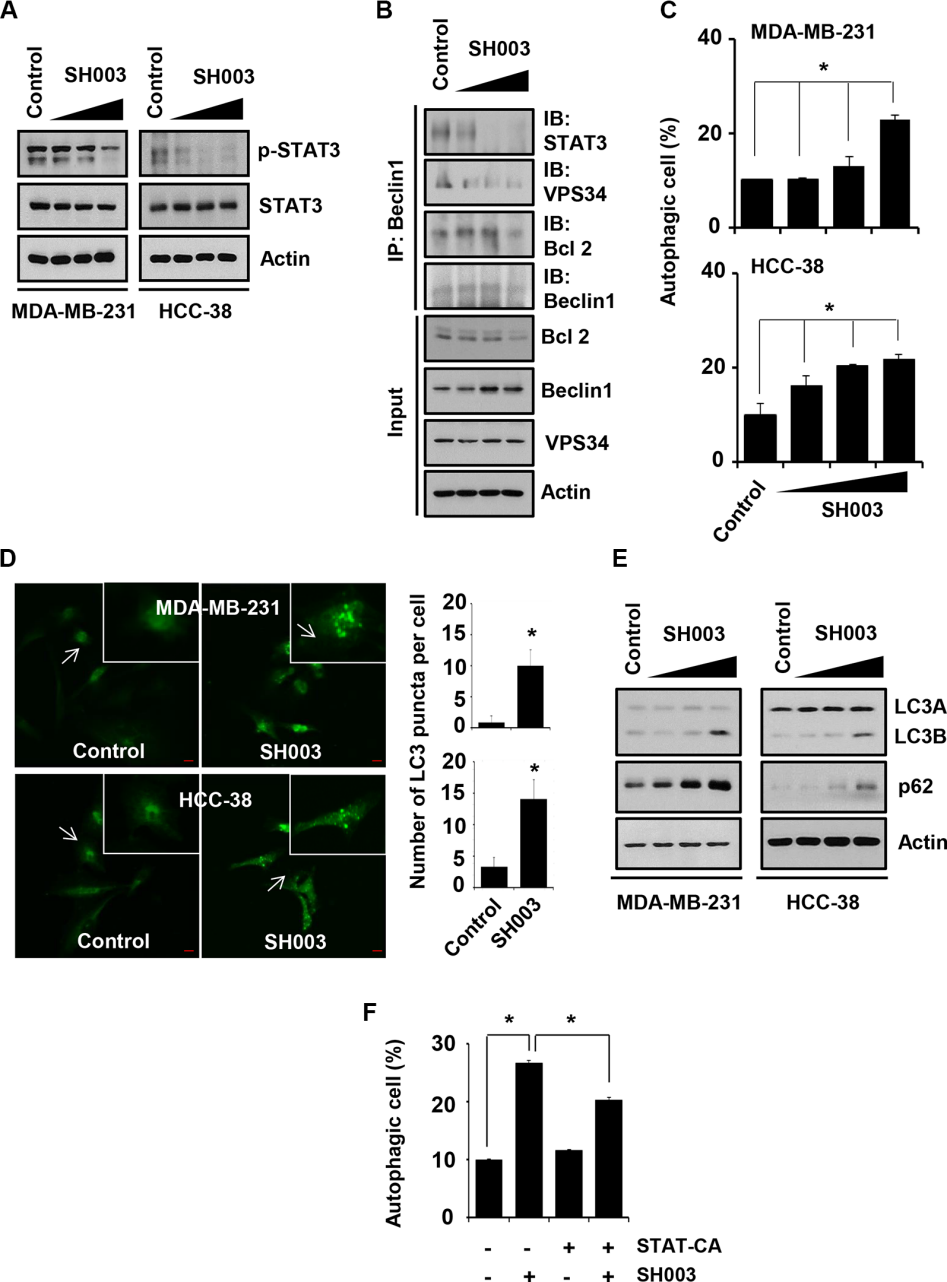
SH003 induces autophagy by suppressing STAT3 phosphorylation (**A**) MDA-MB-231 and HCC-38 cells were treated with different doses of SH003 for 15 minutes and then performed western blots with anti-p-STAT3 and STAT3. Actin was used for the internal control. (**B**) Cells were treated with SH003 for 24 hours and whole-cell lysates were immunoprecipitated with anti-Beclin1 antibody. The immunoprecipitants and input proteins were then blotted with the antibodies for STAT3, VPS34, BCl-2, Beclin1 and actin. (**C**) MDA-MB-231 and HCC-38 cells were treated with SH003 (0, 100, 250 and 500 μg/ml) for 24 hours and then stained with Cyto-ID fluorescence dye for 30 minutes at room temperature in the dark. Data analyzed using a FACSCalibur. Data were analyzed by ANOVA with *P* < 0.05. (**D**) MDA-MB-231 and HCC-38 cells were treated with 500 μg/ml of SH003 for 24 hours and then stained with anti-LC3B antibody (1 μg/ml) and anti-Alexa Fluor-488 (1:250) antibody. LC3 punctate in the cells were analyzed using Olympus FV10i Self Contained Confocal Laser System. The object was 20× and scale bar indicates 10 μm. *P < 0.05. (**E**) Analysis of autophagy-related molecules. Cells were treated with SH003 for 24 hours and whole-cell lysates were analyzed by western blots with anti-LC3A/B and p62/SQSTM1. Actin was used for the loading control. (**F**) Cells were transfected with STAT3-CA and treated with SH003 for 24 hours. Autophagosome formation was stained with Cyto-ID fluorescence. *P < 0.05. Experiments were performed in triplicate. Bars indicate means that standard deviations (SD).

Our previous study found that SH003 targeted STAT3 in breast cancer cells [21]. Consistently, our present study confirmed SH003 inhibition of STAT3 phosphorylation in different breast cancer cells (Figure 2A). STAT3 has been known to regulate autophagy, which is crucial for cancer development [28–30]. We further examined whether SH003 affects autophagy by altering beclin1 interaction with STAT3 and autophagy associated proteins including VPS34 and Bcl-2. In our beclin1 immunoprecipitation assays, SH003 reduced beclin1 interaction with STAT3, VPS34 and Bcl-2 (Figure 2B), suggesting that SH003 might affect autophagy via disrupting beclin1 interaction with STAT3 and autophagy-associated proteins. Therefore, we further examined whether SH003 affect autophagy. SH003 induced autophagy, when cells were treated with SH003 for 24 hours and then stained with Cyto-ID fluorescence dye (a marker for autophagic vacuoles [31]) (Figure 2C). Supportively, SH003 increased the number of LC3 puncta per cell (Figure 2D) and altered LC3A/B ratio (Figure 2E). However, we unexpectedly found that SH003 increased a level of p62 (Figure 2E), while it has been revealed that autolysosome-dependent degradation system regulates p62 level [13, 32]. Thus, those data suggested that SH003 might induce autophagy with no effect on later stages such as autolysosome formation or autolysosomal degradation system. Moreover, constitutively active STAT3 (STAT3-CA) partly rescued that SH003 effect on autophagy (Figure 2F). Therefore, our data indicated that SH003 might induce autophagy by inhibiting STAT3 activation.

### SH003 induces autolysosome formation

**Figure 3:**
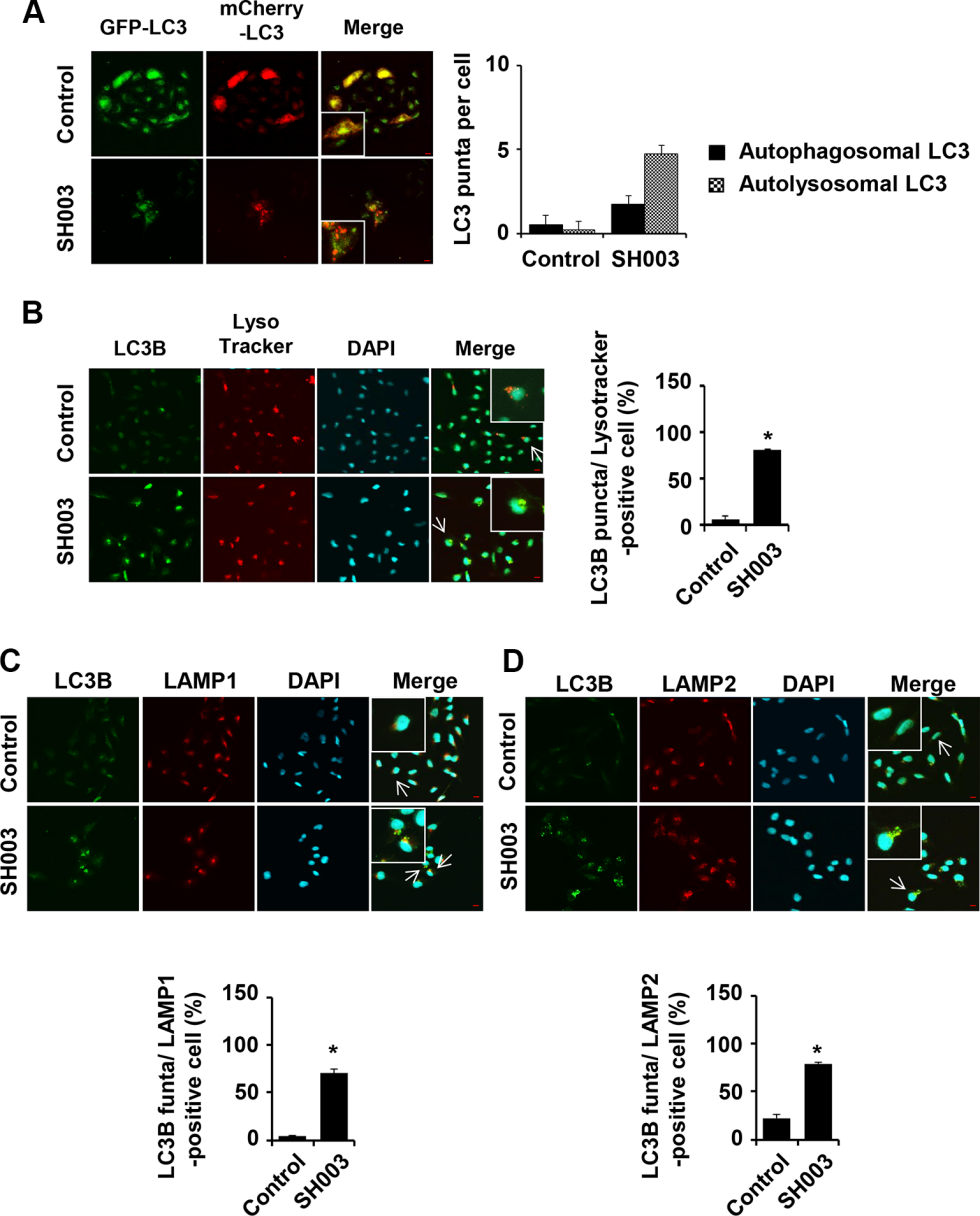
SH003 induces autolysosome formation (**A**) Stable expression of mCherry-GFP-LC3 MDA-MB-231 cells were treated with 500 μg/ml of SH003 for 24 hours and images were obtained with using Olympus FV10i Self Contained Confocal Laser System. Yellow (double staining with GFP and RFP) and red (staining with only RFP) florescence were stained for autophagosome and autolysosome, respectively. The object was 20× and scale bar indicates 10 μm. *P < 0.05. (**B**) MDA-MB-231 cells were treated with SH003 for 24 hours and stained with DND-99 lysotracker dye (75 nM) for 1 hour at 37°C. After fixation, permeabilization and blocking, cells were stained with anti-LC3B and Alexa-488 antibodies. DAPI was used for nucleus staining. (**C**) Cells were treated with SH003 for 24 hours and stained with anti-LC3B and LAMP1 (1:100) antibodies. (**D**) MDA-MB-231 cells were stained with anti-LC3B and LAMP2 (1:100) antibody. Colocalization with LC3B and LAMP2 was analyzed using Olympus FV10i Self Contained Confocal Laser System. The object was 20× and scale bar indicates 10 μm. *P < 0.05.

To test our hypothesis that SH003 induces autophagy with halting either autolysosome formation or autolysosomal degradation system, autosomal vacuoles were distinguished in terms of GFP-mCherry-LC3B vector system. In this system, autophagosomes are visualized in yellow as they express both GFP and mCherry. Autolysosomes are colored in red as those express mCherry alone. SH003 increased numbers of both autophagosomes and autolysosomes (Figure 3A). Lysotracker red is a fluorescent dye that selectively accumulates in acidic vesicles such as lysosomes [33]. SH003 also induced co-localization of LC3B with lysotracker red (Figure 3B). Likewise, SH003 increased LC3B co-localization with lysosomal membrane protein, LAMP1 or LAMP2 (Figure 3C and 3D). Therefore, we concluded that SH003 induced autophagy but might affect autolysosomal degradation system, since it rather increased p62 level.

### Rapamycin enhances SH003 effect on autophagy-mediated apoptosis

**Figure 4:**
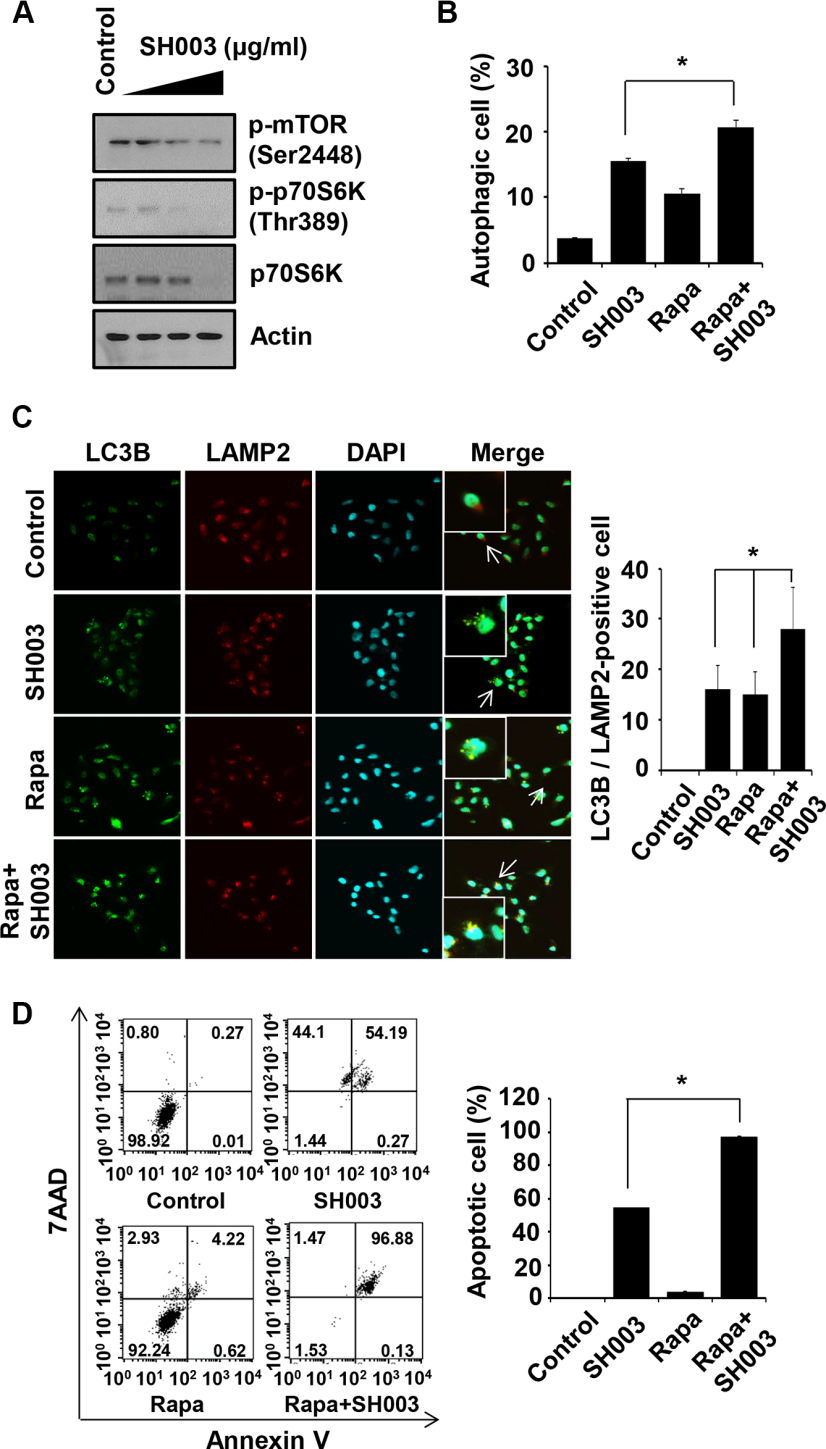
Rapamycin enhances SH003-induced autophagy-mediated apoptosis (**A**) MDA-MB-231 cells were treated with different concentrations of SH003 (0, 100, 250 and 500 μg/ml) for 24 hours and then subjected to western blots with the antibodies indicated (anti-p-mTOR, -p-p70S6K and -p70S6K). Actin was used as internal control. (**B**) Cells were treated with 10 μM of rapamycin (Rapa) and 500 μg/ml of SH003 and then autophagosome vacuoles were measured by Cyto-ID fluorescence. Data analyzed using a FACSCalibur. *P < 0.05. (**C**) MDA-MB-231 cells were treated with rapamycin and SH003 and then stained with anti-LC3B and LAMP2 antibodies. Colocalization with LC3B and LAMP2 were analyzed using Olympus FV10i Self Contained Confocal Laser System. The object was 20× and scale bar indicates 10 μm. *P < 0.05. (**D**) Cells were treated with rapamycin and SH003 for 48 hours and then stained with Annexin V and 7AAD at room temperature in the dark. Annexin V-positive apoptotic cells were detected using FACSCalibur. *P < 0.05. Representative data were presented as the means and standard deviations (SD).

SH003 reduced phosphorylation of mTOR (major repressor autophagy [34]) and p70S6K (mTOR downstream target [35]) (Figure 4A). Moreover, autophagy was more increased when cells were treated with both SH003 and rapamycin (Figure 4B). Consistently, SH003 with rapamycin strongly increased autolysosome numbers (Figure 4C). Moreover, SH003 with rapamycin strongly induced apoptosis (Figure 4D). Those data support our finding that SH003 induces autophagy, and suggest that SH003 may affect other signaling pathways beyond rapamycin target, mTOR-mediated signaling.

### SH003-mediated p62 accumulation via reduction of Cathepsin level in autolysosomes causes autophagy-mediated apoptosis

**Figure 5:**
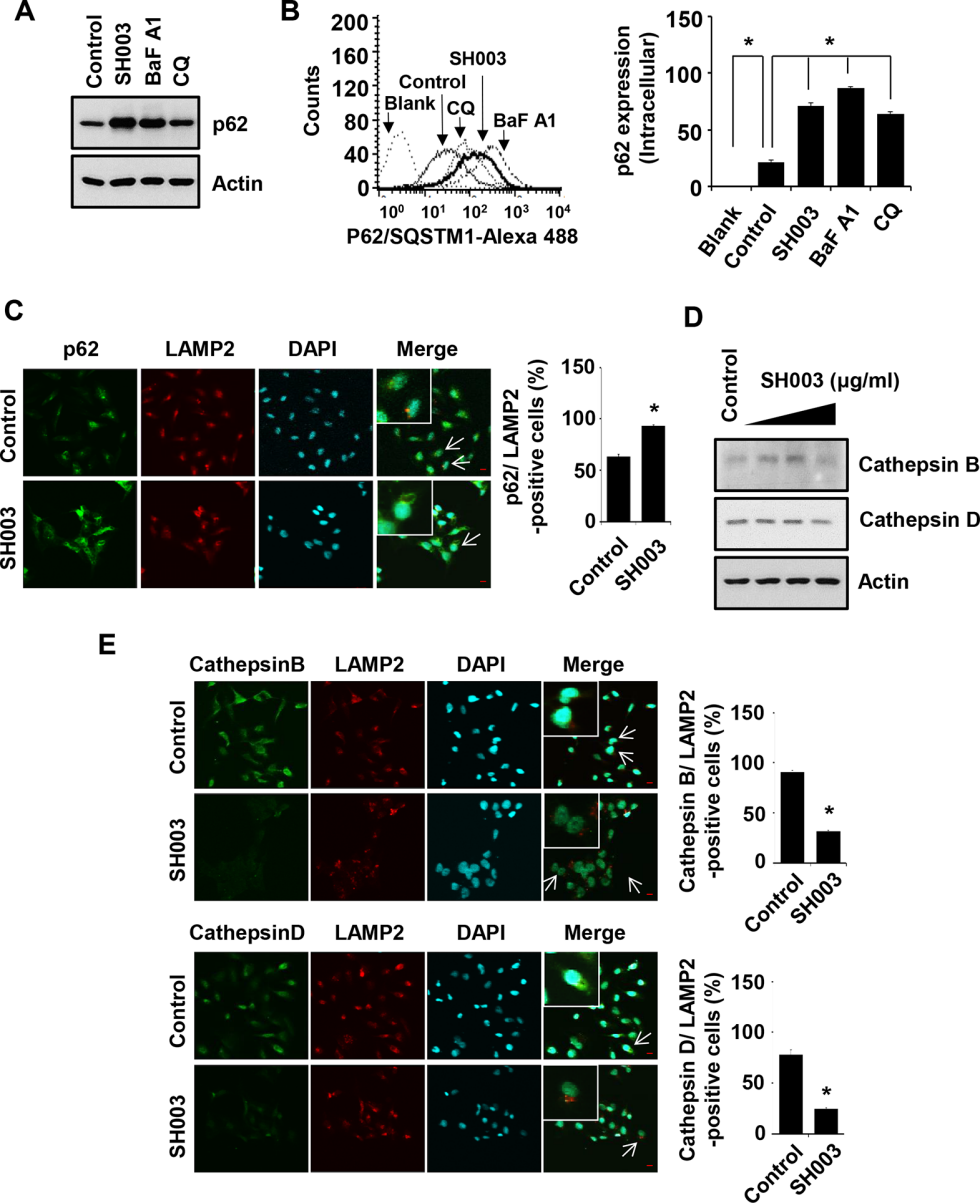
SH003 induces p62 accumulation via reduction of Cathepsin expression (**A**) MDA-MB-231 cells were treated with SH003 (500 μg/ml), BaFA1 (400 nM) and CQ (10 μM) for 24 hours. p62 protein expression was objected with western blots. Actin was used for the internal control. (**B**) Cells were treated with SH003 and autophagy inhibitors (BaFA1 and CQ) for 24 hours and stained with p62 -Alexa 488-conjugated p62 antibody for 30 minutes. p62 accumulation in the cells were detected using FACSCalibur. *P < 0.05. (**C**) MDA-MB-231 cells were treated with SH003 for 24 hours and stained with p62 (1 μg/ml) and LAMP2. DAPI was used as nucleus staining. The object was 20× and scale bar indicates 10 μm. *P < 0.05. (**D**) Cells were treated with SH003 for 24 hours and then performed western blots with anti-Cathepsin B and -Cathepsin D. Actin was used for loading control. (**E**) MDA-MB-231 cells were treated with 500 μg/ml of SH003 for 24 hours and stained with Cathepsin B (1:50)/LAMP2 and Cathepsin D (1:50)/LAMP2. Images were obtained with using Olympys FV10i Self Contained Confocal Laser System. The object was 20× and scale bar indicates 10 μm. *P < 0.05. Experiments were performed in triplicate. Bars indicate means that standard deviations (SD).

p62, a specific substrate of autophagy, is degraded in autolysosomes. Cells were treated with SH003 or autophagy inhibitors such as bafilomycin A1 (vacuolar-type H (+)-ATPase inhibitor [36]) and chloroquine (lysosomal lumen alkalizer [36]), and then p62 level was analyzed using western blots and FACS. SH003 and autophagy inhibitors increased intracellular p62 level (Figure 5A and 5B), suggesting that SH003 accumulation of p62 level might reflect the inhibition of particular step in autophagy processes. Accordingly, those data support our hypothesis that SH003 might inhibit autolysosomal degradation of p62. Supportively, SH003 increased a number of cells where p62 co-localized with LAMP2, indicating that the accumulation of p62 in autolysosomes (Figure 5C). Lysosomal proteases like Cathepsins degrades p62 [1, 37]. SH003 reduced levels of both Cathepsin B and Cathepsin D (Figure 5D). Moreover, SH003 decreased levels of lysosomal Cathepsins (Figure 5E). Thus, those data indicate that SH003 accumulates p62 in autolysosomes by reducing Cathepsins.

**Figure 6:**
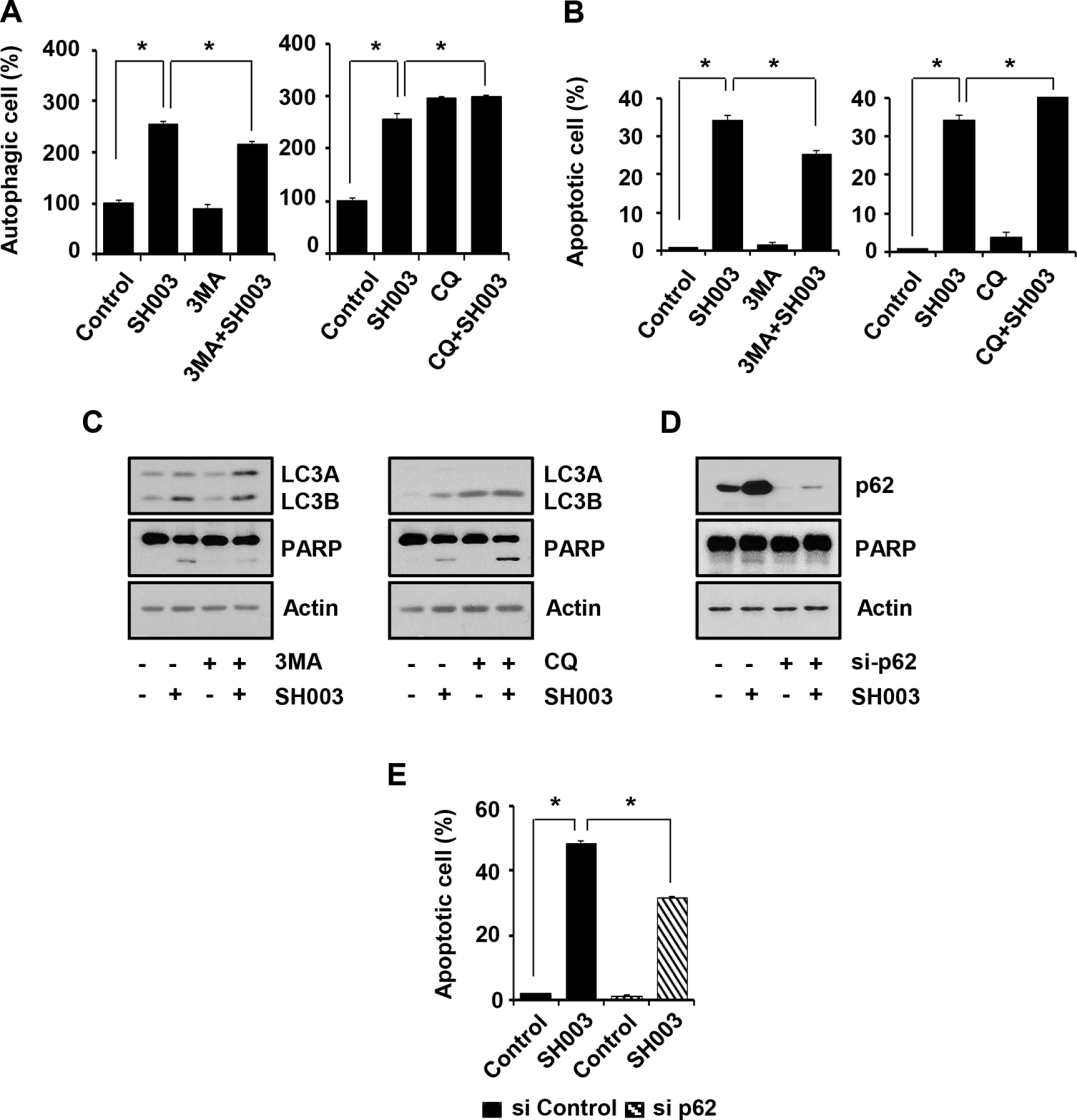
SH003-induced p62 accumulation causes autophagy-mediated apoptosis (**A**) MDA-MB-231 cells were pretreated with 1 mM of 3MA and 10 μM of CQ for 30 minutes and then treated with 500 μg/ml of SH003. After 24 hours, cells were stained with Cyto-ID fluorescence dye for 30 minutes and analyzed using a FACSCalibur. *P < 0.05. (**B**) Cells were treated with SH003 for 24 hours and then stained with annexin V and 7AAD at room temperature in the dark. Autophagy inhibitors (3MA and CQ) were treated 30 minutes before SH003 treatment. Annexin V-positive apoptotic cells were detected using FACSCalibur. *P < 0.05. (**C**) MDA-MB-231 cells were pretreated with 3MA and CQ for 30 minutes and then treated with SH003. 24 hours after treatment, LC3A/B, PARP and actin levels were examined. (**D**) MDA-MB-231 cells were transfected with p62 siRNA, treated with SH003 and performed western blots with anti-p62 and -PARP antibodies. Actin was used as internal controls. (**E**) Cells were transfected with control siRNA and p62 siRNA and then treated with 500 μg/ml of SH003. After 48 hours, cells were stained with Annexin V and 7AAD at room temperature in the dark. Annexin V-positive apoptotic cells were detected using FACSCalibur. *P < 0.05. Experiments were performed in triplicate. Bars indicate means that standard deviations (SD).

Meanwhile, although CQ also accumulated p62 level and induced autophagy, it failed to cause apoptosis (Figures 5A, 5B, 6A and 6B). Therefore, SH003-induced apoptosis might require more complex mechanisms that might not be induced by CQ. Moreover, we found that co-treatment of SH003 with CQ slightly increased cell numbers in autophagy and apoptosis, respectively (Figure 6A and 6B). We confirmed that co-treatment of SH003 with CQ increased PARP cleavage and LC3B production (Figure 6C). Therefore, our data suggested that they could synergize autophagy-induced apoptosis. Furthermore, p62 silencing reduced SH003-mediated apoptosis (Figure 6D and 6E), indicating that p62 might be prominent for SH003-induced apoptosis.

### SH003-induced p62 accumulation causes reactive oxygen species-mediated apoptosis

**Figure 7:**
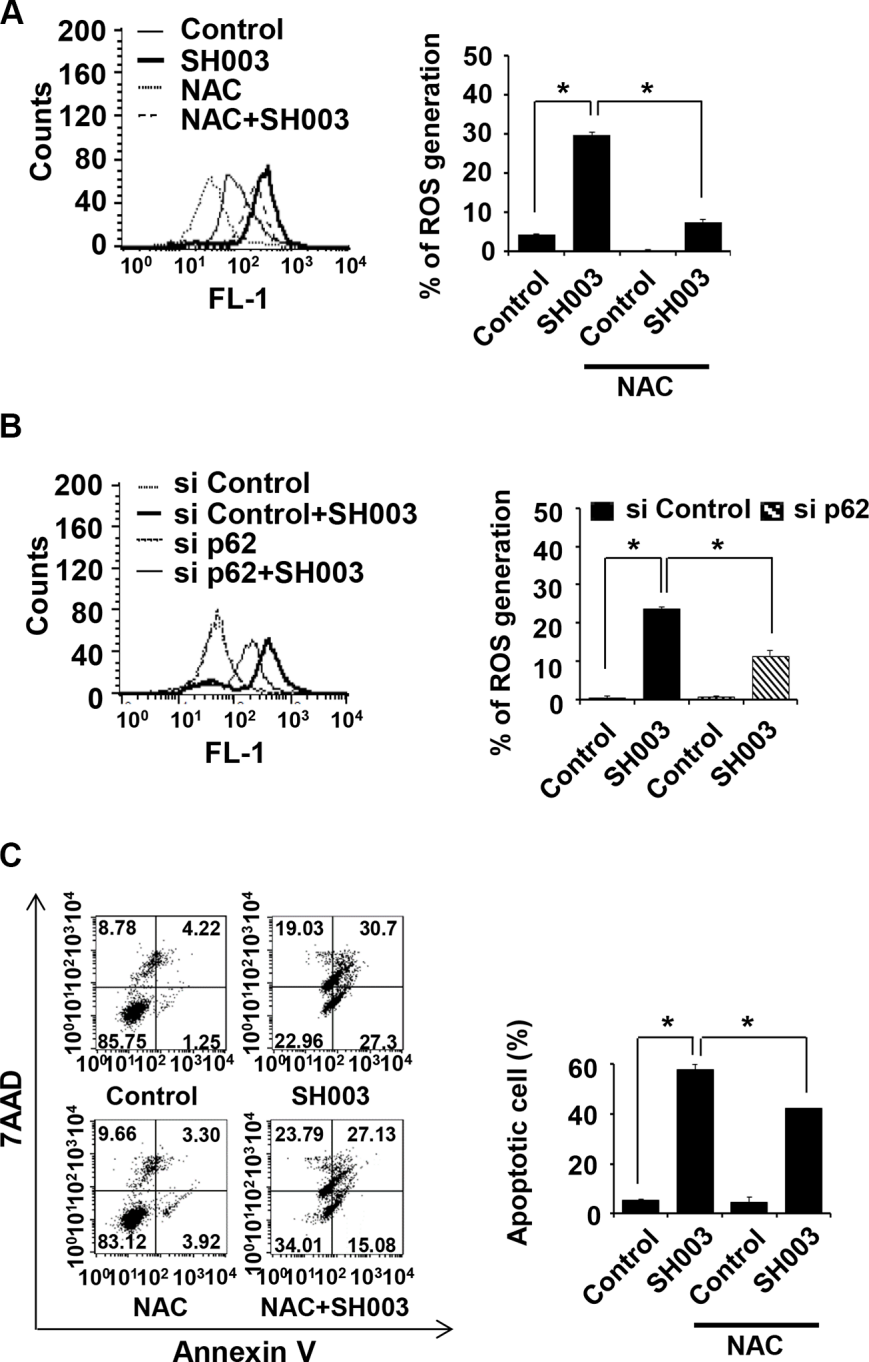
SH003-induced p62 accumulation causes ROS-mediated apoptotic cell death (**A**) MDA-MB-231 cells were pretreated with or without NAC (2.5 mM) for 1 hour, followed by exposure to SH003. After 24 hours, cells were stained with H_2_DCFDA for 1 hour at 37°C. ROS generation was detected with using FACSCalibur by the FL1 channel. (**B**) Cells were transfected with control siRNA and p62 siRNA and then treated with SH003 for 24 hours. ROS generation was measured by FACSCalibur. *P < 0.05. (**C**) Cells were pretreated with NAC for 1 hour and then treated with 500 μg/ml of SH003 for 48 hours. Cells were stained with annexin V and 7AAD. *P < 0.05. Experiments were performed in triplicate. Bars indicate means that standard deviations (SD).

p62 accumulation has been revealed to cause reactive oxygen species (ROS) generation[38–40]. In our study, SH003 increased ROS generation by approximately seven folds, and a well-known ROS scavenger, N-acetyl-Lcysteine (NAC) inhibited SH003 effect on ROS generation (Figure 7A). Moreover, p62 silencing decreased SH003-induced ROS generation (Figure 7B), suggesting that p62 accumulation might be required for ROS generation. In additions, NAC reduced SH003-mediated apoptosis (Figure 7C). Therefore, we draw that SH003-induced p62 accumulation causes ROS-mediated apoptosis.

### SH003 represses *in vivo* tumor growth

**Figure 8:**
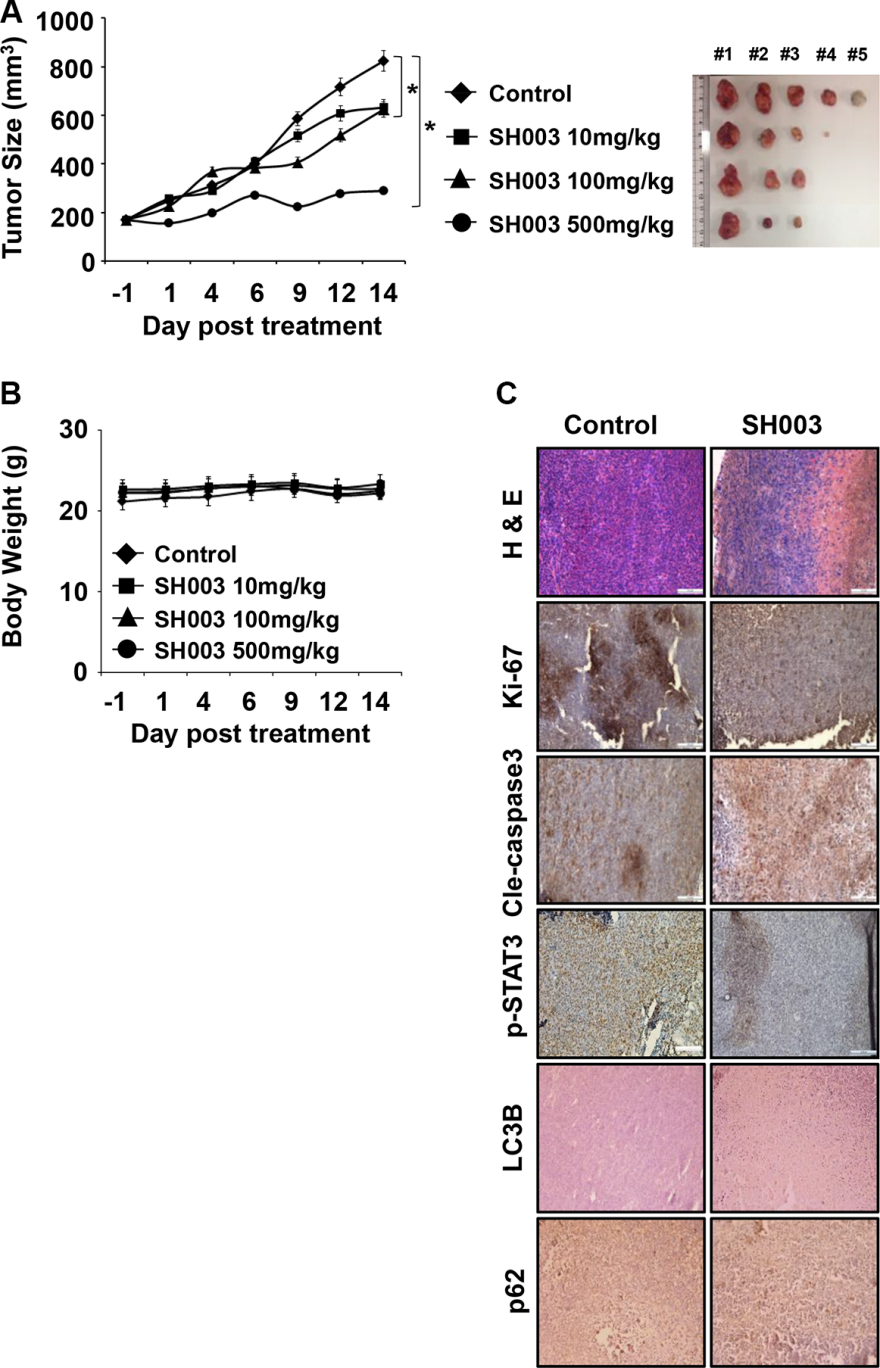
SH003 suppresses *in vivo* tumor growth (**A**) 5 × 10^5^ MDA-MB-231 cells were s.c. injected and mice (*n* = 5/group) were p.o. administrated daily with different concentrations of SH003 (10, 100 and 500 mg/kg) for 15 days. Xenograft tumor volumes and body weight of mice were measured three times a week. Tumor volumes were calculated using the following: Tumor volume (cubic millimeters) = width^2^ × length/2. Data were analyzed by ANOVA with *P* < 0.05. (**B**) Body weight (**C**) Tumor tissues were stained with hematoxylin, eosin and antibodies indicated (anti-Ki-67, -Cleaved caspase 3, -p-STAT3, -LC3B and -p62). Images were obtained at 20× magnification. The scale bar indicates 100 μm. Bars indicate means that standard deviations (SD).

To examine SH003 effect *in vivo*, we conducted the xenograft mouse tumor growth assay by orthotopically injecting MDA-MB-231 cells into nude mice. Mice were then orally administrated with different concentrations (0, 10, 100 and 500 mg/kg) of SH003 every day for 15 days. SH003 at 500 mg/kg strongly inhibited tumor growth with no effect on body weight (Figure 8A and 8B). In addition, our histology data showed that SH003 decreased Ki-67 and p-STAT3-positive cell numbers and increased the number of cells stained with cleavage Caspase-3, LC3B or p62 (Figure 8C). Thus, our *in vivo* study confirmed that SH003 suppresses tumor growth by inducing autophagy-mediated apoptosis.

### SH003 toxicity

We SH003 toxicity tests in Sprague-Dawley (SD) rats. To test an acute toxicity, the animals were divided into 4 groups: a vehicle control group and SH003-administrated groups (500, 1000 and 2000 mg/kg), each group was consisted of 5 rats of each sex. The rats were orally administrated with distilled water (vehicle group) or SH003. Fourteen days after oral administration, body weight, mortality, clinical signs and gross findings were recorded (Figure S1 and Table S1). Thus our acute toxicity data suggest that the lethal dose was more than 2000 mg/kg for both genders. For four-week-repeated oral dose toxicity study, animals were orally administrated with SH003 at different doses (0, 500, 1000 and 2000 mg/kg) every day for 4 weeks. SH003 did not result in any toxicological changes, such as mortality, common symptom, body weight, food intake, hematological values, serum biochemical values, relative organ weights, clinical signs and histopathology (Figure S2 and Table S2). Considering our toxicity studies, the no-observed-adverse-effect level (NOAEL) was determined higher than 2000 mg/kg for both male and female animals. In thirteen-week-repeated oral dose toxicity test, male and female rats were randomly assigned to four groups: control (*n* = 15), low (625 mg/kg SH003, *n* = 10), medium (1250 mg/kg, *n* = 10) and high (2500 mg/kg, *n* = 15) groups. The rats were orally administrated with distilled water (vehicle group) or SH003 every day. Thirteen weeks after administrations, we allocated five of both male and female rats from the control and 2500 mg/kg SH003-treated group, respectively, and observed those animals for another four weeks. SH003 resulted in no significant differences in body weight changes, food consumptions, ophthalmoscopy findings, urinalysis, hematological values, serum biochemical values, blood coagulation values, absolute organ weights and clinical signs. In additions, hypertrophy of liver was observed in the 13-week repeated toxicity test, but this symptom was not observed during the 4-week recovery period (Figure S3 and Table S3). Thus our 13-week repeated-with a 4 weeks recovery period data demonstrated that NOAEL was more than 2500 mg/kg for both genders.

### SH003 component profiling and mechanism action

Components in SH003 were analyzed using high performance liquid chromatography (HPLC), and characterized in terms of both retention time and UV spectrum. This analysis confirmed twenty components (tryptophan: tR 2.93 min, umbeliferone: tR 3.20 min, chlorogenic acid: tR 3.76 min, 3-O-feruloylquinic acid: tR 7.20 min, calycosin-7-O-β-D-glucoside: tR 8.44 min, nodakenin isomer: tR 9.01 min, nodakenin: tR 9.08 min, marmesin / 7-hydroxy-6-(2R)-hydroxy-3-methylbut-3-ethyl)coumarin: tR 10.17 min, marmesin / 7-hydroxy-6-(2R)-hydroxy-3-methylbut-3-ethyl)coumarin: tR 11.21 min, decursinol: tR 11.75 min, calycosin: tR 12.04 min, bergapten(5-methoxypsoralen) : tR 13.79 min, isopimpinellin: tR 14.90 min, formononetin: tR 15.05 min, (6aR, 11aR)-3-hydroxy-9,10-dimethoxypterocarpa: tR 15.47 min, osthenol: tR 17.60 min, decursin: tR 20.40 min, decursinol angelate : tR 20.57 min and two-unknown: tR 1.59 min and 20.85min). In addition, one peak at 11.1 minutes (formononetin-7-O-β-D-glucoside) was not detected in SH003, while being found in Am (Figure S4 and S5). We recently confirmed SH003-contained components, such as formononectin, decursin, nodakenin and curcubitacin D [21]. Therefore, we further examined whether effects of these compounds were similar to those of SH003 in cancer cells. Cucurbitacin D showed similar effects to SH003 on autophagy and apoptotic cell death, while formononectin, decursin and nodakenin did not. Therefore, cucurbitacin D might be one of effective components of SH003 (Figure S6).

### Herb-drug interaction

We further evaluated CYP450-mediated dug metabolism to test herb-drug interactions. Human liver microsomes were preincubated with different doses of SH003 (1, 3, 10, 30, 100 and 300 μg/ml), and then added with the substrates (40 μm of phenacetin, 2.5 μm of coumarin, 10 μm of paclitaxel, 10 μm of diclofenac, 160 μm of (±)-mephenytoin, 5 μm of dextromethorphan and 2.5 μm midazolam). SH003 had minimal inhibitory effects on all CYP isozymes and its IC50 was considered higher than 300 μg/ml, although it reduced CYP1A2 enzyme activity with no significance (Table S4).

## DISCUSSION

Our previous reports have shown that SH003 compared to each herbal component extract (Am, Ag and Tk) inhibited tumor growth and metastasis on highly metastatic MDA-MB-231 breast cancer cells, both *in vivo* and *in vitro* without toxicity [21]. Moreover, SH003 regulation of STAT3 signaling was crucial for the inhibition of cancer growth and metastasis. We confirmed SH003 inhibition of tumor growth by repressing STAT3 activation. As SH003 caused apoptotic cell deaths of both MDA-MB-231 and HCC-38 breast cancer cells, it is convincing that SH003 could treat highly metastatic breast cancer. In molecular and cellular mechanisms, SH003 altered STAT3 interaction with Beclin1/VPS34/Bcl2 complexes. Moreover, sustained STAT3 activation reduced SH003 induction of autophagy. Autophagy induction requires STAT3 interaction with Beclin1/VPS34/Bcl2 complexes. Thus, SH003 regulation of STAT3 activation and complex formation would be crucial for the induction of autophagy and cancer treatment. In addition, we found that SH003 inhibited the activation of mTOR signaling in the induction of autophagy, which is consistent with recent studies that showed the inhibition of mTOR-STAT3 in autophagy induction [41, 42]. Our data further suggest that mTOR and STAT3 might regulate autophagy in two different ways, as SH003 and rapamycin showed synergistic effect in autophagy induction. While we know mTOR and STAT3 regulate autophagy in the same autophagy signaling circuit [43–45], it is unclear whether and how mTOR and STAT3 independently works in autophagy induction. Thus, we need more basic knowledge to understand autophagy in treatment of cancer disease.

**Figure 9:**
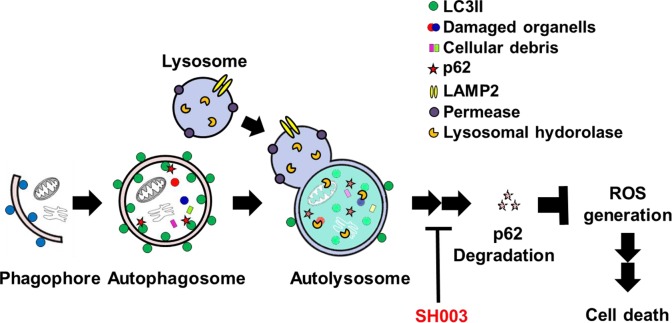
A schematic representation of the mechanisms for SH003 suppression of breast cancer growth

We further found that SH003 induction of autophagy increased p62 level, although autophagy has been known to reduce p62 level [32, 46]. Furthermore, SH003 reduced protein levels of Cathepsins, while it increased autolysosome numbers. SH003 induction of autophagy disrupts p62 degradation, resulting in apoptotic cell death via altering intracellular ROS level. Therefore, our data suggest that SH003 inhibition of p62 degradation in autolysosome causes ROS-mediated apoptotic cell death (Figure 9). Recent studies revealed that STAT3 regulates gene expression of Cathepsins [47]. Thus, SH003 inhibition of mTOR-STAT3 appears to increase p62 level by downregulating expression of Cathepsins.

Our study shows that SH003 inhibits cancer growth both *in vitro* and *in vivo* systems via inductions of autophagy-mediated apoptosis. Moreover, studies using herbal medicine reveal one of biological mechanisms of autophagy-mediated apoptosis. However, it is still unclear which chemical components directly affect mTOR and/or STAT3. Therefore, our ongoing studies hope to decipher how chemical components of SH003 work biochemically in the cells. Meanwhile, toxicity studies with GLP regulations conclude that SH003 is safe in rats. Thus, our future clinical studies will answer whether SH003 can be used to treat cancer with safety.

## MATERIALS AND METHODS

### Chemicals and reagents

Cyto-ID autophagy detection kit was obtained from Enzo Life Sciences (Villeurbanne, France). Live and dead cell assay kit was purchased from Abcam (Cambridge, UK). 3-(4,5-dimethylthiazol-2-yl)-2,5-diphenyl-tetrazolium bromide (MTT), bafilomycin A1 (BaF A1), chloroquine (CQ), 3-methyladenine (3-MA) formononectin, decursin and N-acetyl-L-cysteine (NAC) were from Sigma-Aldrich (St. Louis, MO, USA). Alexa fluor-488, Alexa fluor-594 and Lipofectamine reagent were obtained from Invitrogen (Garlsbad, CA, USA). Protein A/G plus-agarose beads and p62 siRNA were purchased cell signaling (Danvers, MA, USA). pBABE-puromCherry-EGFP-LC3B (addgene 22418) and EF.STAT3C.Ubc.GFP (addgene 24983) were from addgene (Cambridge, MA, USA). Nodakenin was purchased from ChemFaces (Wuhan, China) and cucurbitacin D was obtained from Extrasynthese (Genay, France).

### Preparation of SH003 and HPLC analysis

SH003 extract was provide from Hanpoong Pharm and Foods Company (Jeonju, Republic of Korea) manufactured by the Good Manufacturing Product (GMP). Extraction procedures were reported in previous study [21].

### Cell line and cell culture

Human MDA-MB-231 cells were provided from American Type Culture Collection (Rockville, Maryland). The HCC-38 cells were obtained from the Korean Cell Line Bank (Seoul, South Korea). MDA-MB-231 cells were grown in DMEM medium containing 10% FBS and antibiotics. HCC-38 cells were cultured in RPMI-1640 medium with 10% fetal bovine serum (FBS) 1% antibiotics.

### Cell viability and apoptotic analysis

Cells were seed in 96-well plates and treated with different doses of SH003 for 48 hours. Cell viability was determined using the MTT assay and absorbance was read at 570 nm on the ELISA reader (Molecular Devices, Palo Alto, CA, USA). Cells were seed in 60 mm dishes and treated with SH003 for 48 hours. Cells were harvested, resuspended in binding buffer and stained with Annexin V-FITC and 7-AAD in the dark at room temperature for 15 minutes. Annexin-positive apoptotic cells were measured by FACScalibur (BD Biosciences, San Jose, CA, USA). Live and dead assay was performed with the live and dead cell assay kit according to the manufacturer’s instruction.

### Western blot and measurement of intracellular p62 by flow cytometry

Cells were seed in 6-well plates and treated with different doses of SH003 for 24 hours. Cells were lyzed with RIPA buffer and equal amount of protein (15 μg) in total cell extracts was separated by SDS-PAGE. After transferring to PVDF membrane, the membrane was blocked and blotted with the relevant primary antibodies. Anti-Bax, -Bcl2, -LC3A/B, Cathepsin B, Cathepsin D and -actin antibodies were purchased from Santa Cruz Biotechnology (Santa Cruz, CA, USA). Anti-cleaved caspase-3, -PARP, -p-mTOR, -p-p70S6K and -p70S6K antibodies purchased from Cell Signaling (Danvers, MA, USA). Anti-LC3B and -p62 antibodies were purchased from Abcam (Cambridge, UK). Analysis of intracellular p62 expression was by flow cytometry using the Alexa Fluor 488-conjugated p62 antibody (BD Biosciences, San Jose CA, USA). Cells were seed in 6-well plates and then treated with 500 μg/ml of SH003 and autophagy inhibitors, such as BaF1 and CQ for 24 hours. After permeabilized with 0.5% Tween-20 in 95% ethanol for 10 minutes, stained with Alexa Fluor 488-conjugated p62 antibody (1:50) for 30 minutes in dark. The data was analyzed by FACSCalibur flow cytometry measuring the green signal by the FL1 channel.

### Cyto-ID autophagy detection assay and ROS measurement

Cyto-ID autophagy detection kit measures autophagic vacuoles by flow cytometry. Cells were seed in 6-well plates and treated with different doses of SH003 for 24 hours and stained with Cyto-ID green dye (1 μl/4 ml assay buffer) for 30 minutes in dark. The data was analyzed by FACSCalibur flow cytometry measuring the green signal by the FL1 channel. For ROS generation, cells were seed in 6-well plates and pretreated with NAC (2.5 mM) for 1 hour before SH003 treatment. After 24 hours, cells were stained with H_2_DCFDA for 1 hour at 37°C and analyzed by FACSCalibur flow cytometry measuring by the FL1 channel.

### Confocal microscopy

For LC3B puncta formation, cells were seed in 6-well plates with coverglasses and treated with SH003 for 24 hours. Cells were stained with anti-LC3B antibody (1 μg/ml) and anti-Alexa Fluor-488 (1:250) antibody. For acidic organelles labeling, cells were incubated with lysotracker dye (Lysotracker Red DND-99, 75 nM) for 1 hour at 37°C and then stained with anti-LC3B antibody. For LAMP2 with p62 and Cathepsins staining, cells were stained with 1 μg/ml of LC3B, 1:100 of LAMP-1 and LAMP-2. Finally for p62/SQSTM1, Cathepsin B/LAMP2 and CathepsinD/LAMP2 staining, cells were stained with 1 μg/ml of p62, 1:100 of Cathepsin B, Cathepsin D and LAMP2. For the counter staining, DAPI was used to stain the nucleus. Images were acquired with Olympus FV10i Self Contained Confocal Laser System.

### Transfection

In autophagosome, because LC3B is located on the membraines of autophagosome, GFP and mCherry co-localize and which can be visualized as a yellow pucta. In additions, only red signal is observed in autolysosome because mCherry retains its signal in the acidic pH but GFP signal is declined [48]. For mCherry-GFP-LC3 detection, expression vectors were transfected into cells by using Lipofectamine reagent. One day after transfection, transfected-cells were selected by using puromycin for 14 days. Stable expression of mCherry-GFP-LC3 cells were seed and treated with SH003 for 24 hours. Images were acquired with Olympus FV10i Self Contained Confocal Laser System. For p62 transient knockdown, cells were seed in 6-well plates and transfected with control and p62 siRNA using Lipofectamine reagent, followed by the Annexin V apoptosis analysis, western blot and ROS generation.

### *In vivo* studies

Animal studies were approved by Kyung Hee University Institutional Animal Care and Use Committee (KHU-IACUC). Six-week-old nude (*Nu/Nu*) mice were obtained from Orient Bio (Seongnam, Korea). Mice were injected s.c with 5 × 10^5^ MDA-MB-231 cells and different dose of SH003 were *p.o* administrated daily for 15 days. Body weights and tumor volumes were monitored three times a week. Especially, tumor volumes were measured using caliper and calculated (width^2^ × length/2). For immunohistochemistry, tumors were removed and fixed with 4% formaldehyde. Tumor tissues were embedded, dissected, diparaffinized and stained with Ki-67, Cle-casplase-3, p-STAT3, LC3B and p62. Images were obtained with bright field microscope. The object was 20× and the scale bar on the image (100 μm).

### Statistics

All the data were performed in triplicate, and shown as means and standard deviations (SD). Data analyzed by Student’s *t*-test or one-way ANOVA.

## SUPPLEMENTARY MATERIALS FIGURES AND TABLES




