# Supplementary material for: SH003 suppresses breast cancer growth by accumulating p62 in autolysosomes
Source: Oncotarget. 2016 Aug 19;8(51):88386–400. doi: 10.18632/oncotarget.11393 (PMC5687613; doi:10.18632/oncotarget.11393)
Supplement: Supplementary file 1 [file oncotarget-08-88386-s001.pdf]

SH003 suppresses breast cancer growth by accumulating p62 in autolysosomes

Supplementary Materials

Extended Experimental Procedures

Toxicity of SH003

To test an acute toxicity, the rats were orally administrated with SH003 (0, 500, 1000 and 2000 mg/kg). After 14 days, body weight, mortality, clinical signs and gross findings were observed. For four-week-repeated oral dose toxicity study, animals were orally administrated with SH003 at different doses (0, 500, 1000 and 2000 mg/kg) every day. Four weeks after administration, body weight, mortality, food intake, common symptom, hematological values, serum biochemical values, relative organ weights, clinical signs and histopathology were recorded. Finally, for thirteen-week-repeated oral dose toxicity test, rates were orally administrated with SH003 (0, 625, 1250 and 2500 mg/kg) every day. Thirteen weeks after administration, we allocated rats from the control and 2500 mg/kg SH003-treated group, and observed for another four weeks. Body weight, food consumptions, ophthalmoscopy finding, urinalysis, hematological values, serum biochemical values, blood coagulation values, absolute organ weights and clinical signs were observed. For histopathological change by SH003, female BALB/c mice were Orient Bio (Seongnam, Korea). 2000 mg/kg of SH003 were *p.o* administrated daily for four weeks. Fixed organs (brain, heart, kidney, liver and lung) were embedded in paraffin and stained with hematoxylin and eosin.

HPLC analysis of SH003

HPLC and UPLC were performed to confirm characteristics of SH003 and its components (Elcom Science, Seoul, Republic of Korea).

Herb-drug interaction

Human liver microsomes were preincubated with SH003 (1, 3, 10, 30, 100 and 300 μg/ml) and the substrates (phenacetin, coumarin, paclitaxel, diclofenac, mephenytoin, dextromethorphan and midazolam were added. After incubation, the reaction was stopped by stop solution.

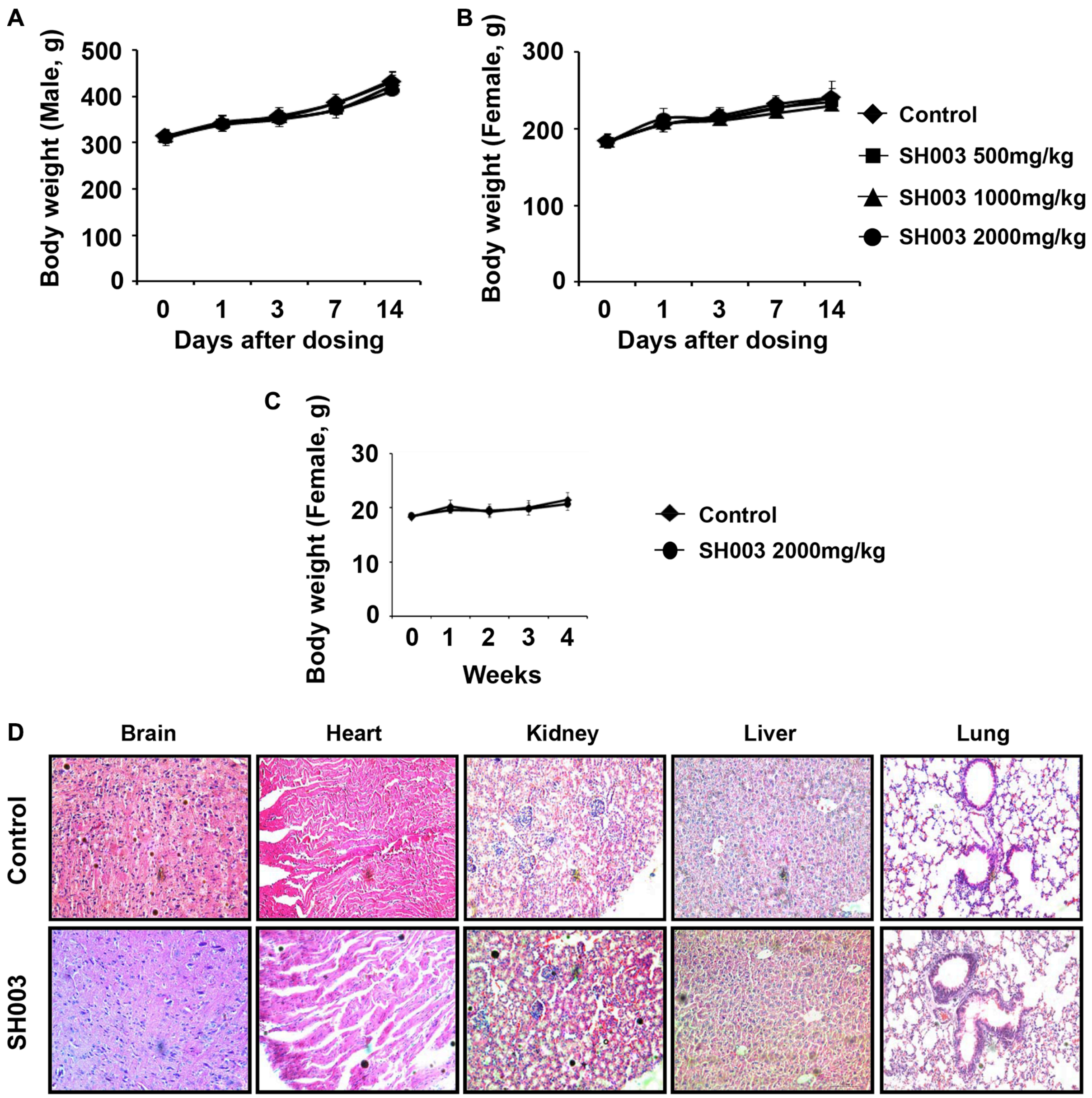

Supplementary Figure S1: Acute toxicity of SH003. Rats (*n* = 5 of male and *n* = 5 of female/group) were administrated with different doses of SH003 (0, 500, 1000 and 2000 mg/kg). During 14 days after acute orally administrated, all animals were observed body weight (**A**) and food intake (**B**). Mice were administrated with 2000 mg/kg of SH003 for four weeks and all mice were observed body weight (**C**). Brain, heart, kidney, liver and lung organs were stained with hematoxylin and eosin for histopathological observations (**D**).

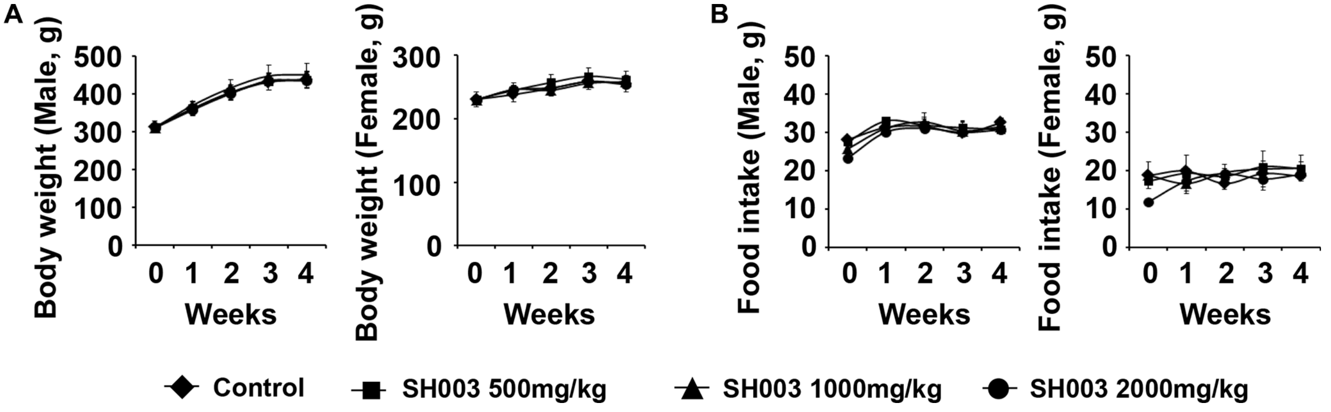

Supplementary Figure S2: Acute toxicity of SH003. Rats (*n* = 5 of male and *n* = 5 of female/group) were administrated with different doses of SH003 (0, 500, 1000 and 2000 mg/kg). During 14 days after acute orally administrated, all animals were observed body weight. (**A**) body weight of male (**B**) body weight of female.

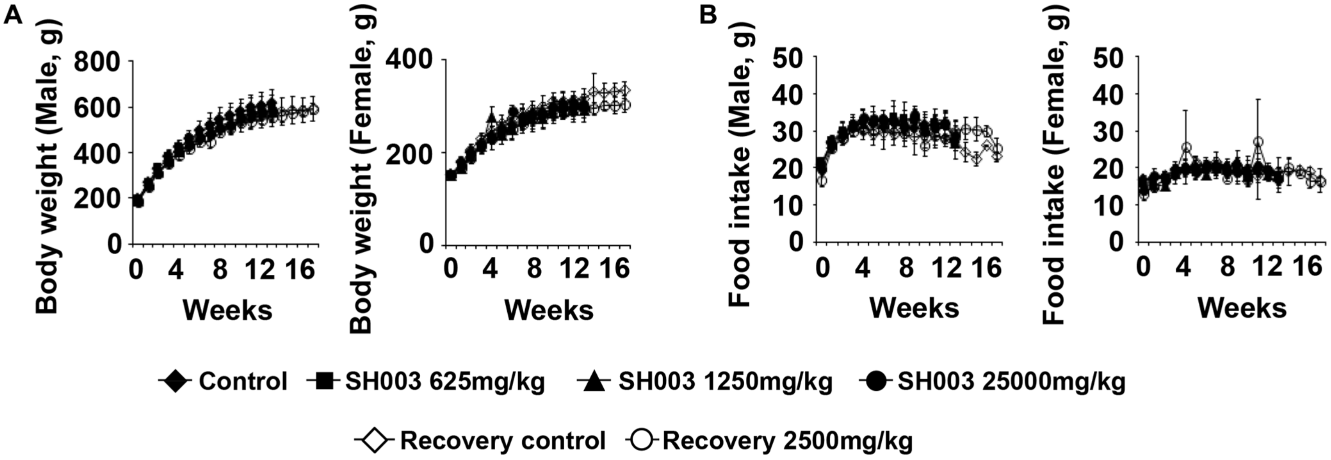

Supplementary Figure S3: 13-week repeated-with a 4-week recovery toxicity of SH003. Male and female rates were divided in to 4 groups: the control (*n* = 15), 62.5 mg/kg of SH003 (*n* = 10), 1250 mg/kg of SH003 (*n* = 10) and 2500 mg/kg of SH003 (*n* = 15). Animals were orally administrated with distilled water or SH003 daily. After 13 weeks, five rats both male and female from the control and 2500 mg/kg of SH003 group were selected and recovered for 4 weeks. All rates were observed body weight (**A**) and food intake (**B**).

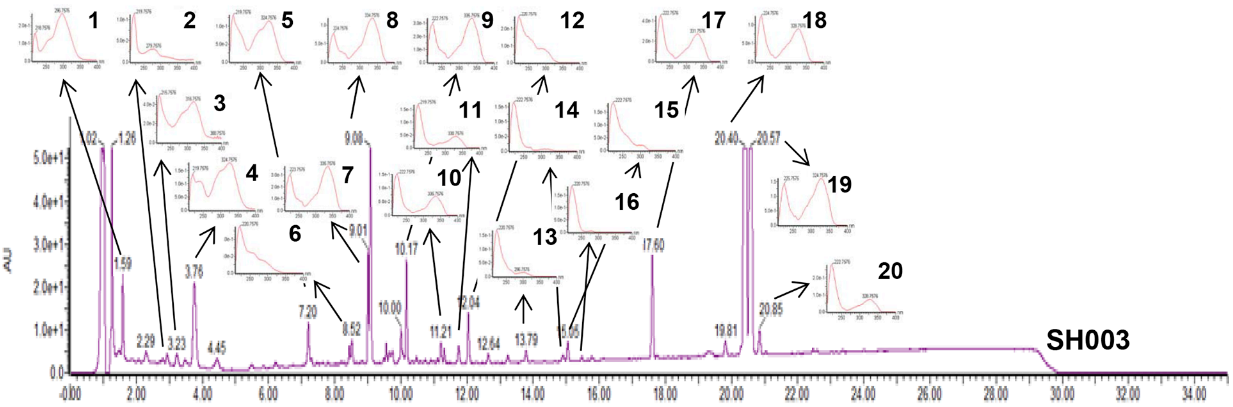

No.	R.T (min)	UV max	Identification	Element compositon
1	1.59	260, 296	unknown	-
2	2.93	279	tryptophan	$C_{11}H_{13}N_2O_2$
3	3.20	285, 316	umbeliferone	$C_9H_7O_3$
4	3.76	245, 296, 324	chlorogenic acid	$C_{16}H_{19}O_9$ / $C_{16}H_{18}O_9Na_1$
5	7.20	240, 296, 324	3-O-feruloylquinic acid	$C_{17}H_{21}O_9$ / $C_{17}H_{20}O_9Na_1$
6	8.44	265, 280	calycosin-7-O-β-D-glucoside	$C_{22}H_{23}O_{10}$
7	9.01	335	nodakenin isomer	$C_{20}H_{25}O_9$ / $C_{40}H_{49}O_{18}$
8	9.08	334	nodakenin	$C_{20}H_{25}O_9$ / $C_{40}H_{49}O_{18}$
9	10.17	335	marmesin / 7-hydroxy-6-(2R)-hydroxy-3-methylbut-3-ethyl)coumarin	$C_{14}H_{15}O_4$
10	11.21	335	marmesin / 7-hydroxy-6-(2R)-hydroxy-3-methylbut-3-ethyl)coumarin	$C_{14}H_{15}O_4$
11	11.75	330	decursinol	$C_{14}H_{15}O_4$
12	12.04	250, 290	calycosin	$C_{16}H_{13}O_5$
13	13.79	296	bergapten(5-methoxypsoralen)	$C_{12}H_9O_4$
14	14.90	310	isopimpinellin	$C_{13}H_{11}O_5$
15	15.05	300	formononetin	$C_{16}H_{13}O_4$
16	15.47	277	(6aR, 11aR)-3-hydroxy-9,10-dimethoxypterocarpan	$C_{17}H_{17}O_5$
17	17.60	331	osthenol	$C_{14}H_{15}O_3$
18	20.40	328	decursin	$C_{19}H_{21}O_5$
19	20.57	324	decursinol angelate	$C_{19}H_{21}O_5$
20	20.85	328	unknown	$C_{19}H_{23}O_5$

Supplementary Figure S4: HPLC profile of SH003. SH003 were analyzed using HPLC and characterized in terms of both retention time and UV spectrum. Twenty components were identified in SH003.

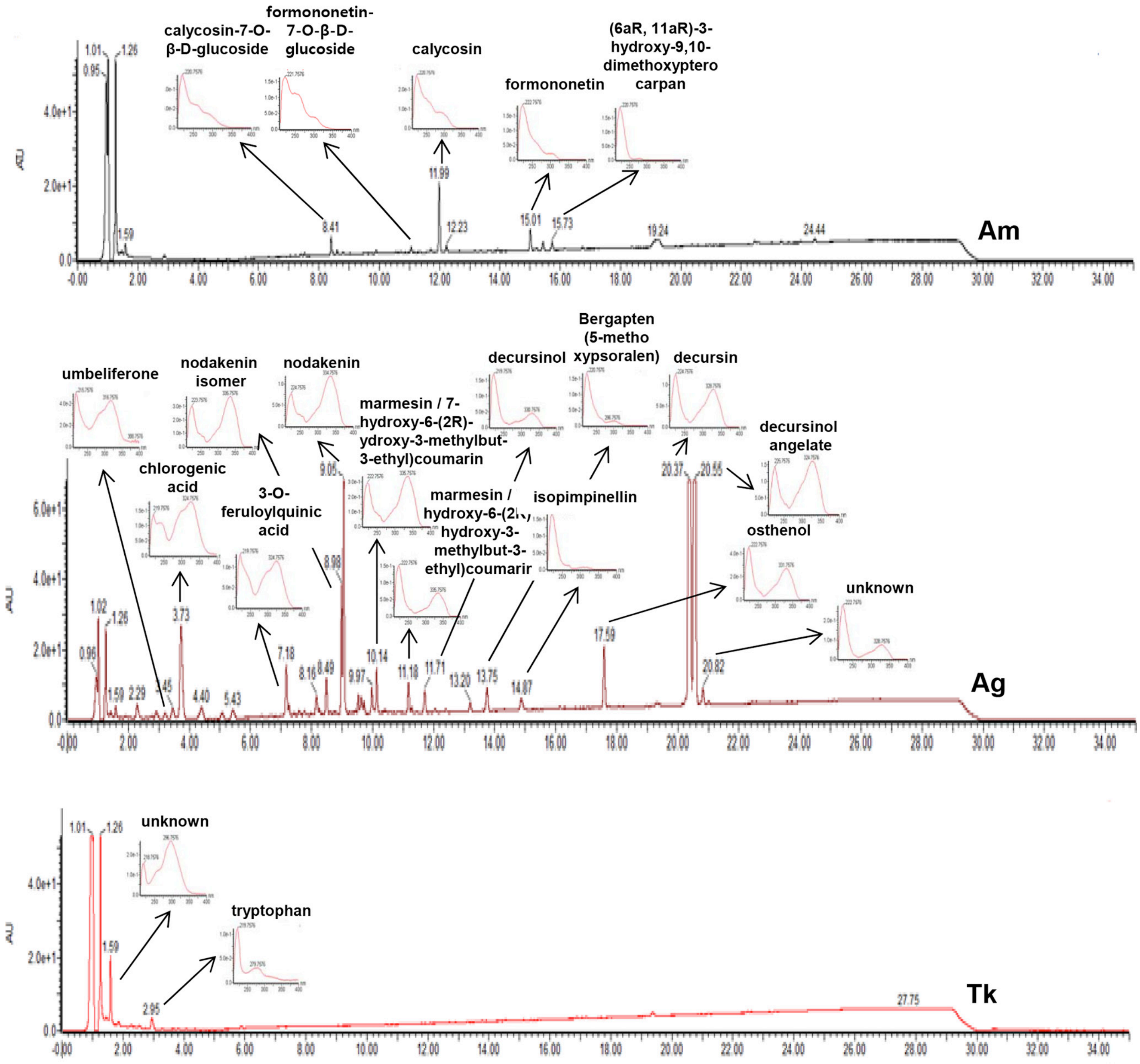

Supplementary Figure S5: HPLC profile of Am, Ag and Tk. SH003 components were analyzed using HPLC and characterized in terms of both retention time and UV spectrum. Five, fourteen and two components were identified in Am, Ag and Tk, respectively.

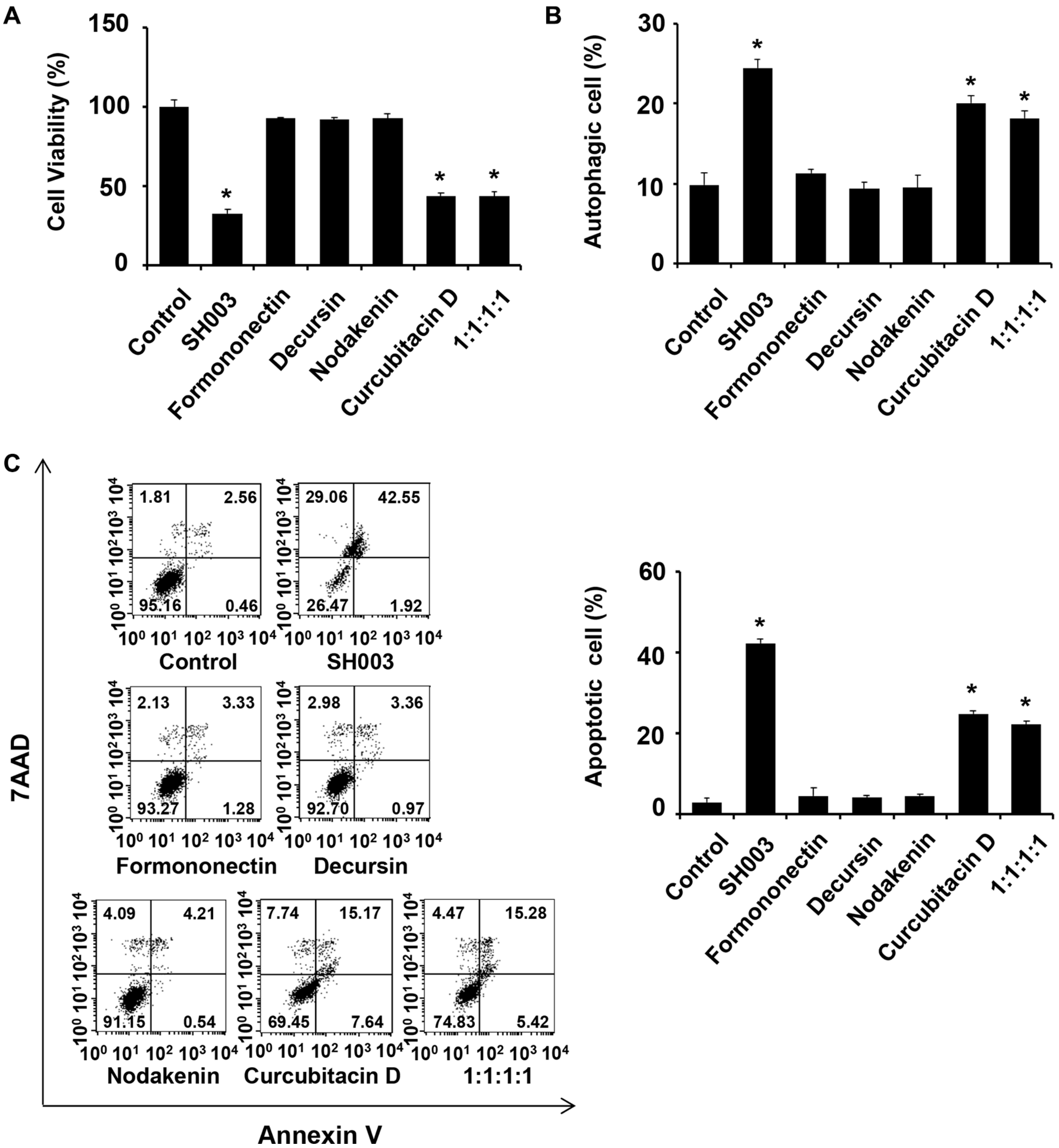

Supplementary Figure S6: Effect of SH003-derived compounds on autophagy and cell death. (**A**) MDA-MB-231 cells were treated with 500 μg/ml of SH003 and 10 μM of compounds (formononectin, decursin, nodakenin and cucurbitacin D) for 24 hours and then stained with Cyto-ID-fluorescence dye for 30 minutes at room temperature in the dark. Data analyzed using a FACSCalibur. **P* < 0.05. (**B** and **C**) Cells were treated with SH003 and compounds for 48 hours and then Annexin V and MTT assay were performed. **P* < 0.05. Experiments were performed in triplicate. Bars indicate means and standard deviations (SD).

Supplementary Table S1: Acute toxicity of SH003

A

Sex	Group (mg/kg)	Dead (*n*)	Survive (*n*)	Mortality (%)
**Male**	**0**	0	5	0
	**500**	0	5	0
	**1000**	0	5	0
	**2000**	0	5	0
**Feale**	**0**	0	5	0
	**500**	0	5	0
	**1000**	0	5	0
	**2000**	0	5	0

B

Sex	Group (mg/kg)	Clinical signs	Gross findings
**Male**	**0**	No abnormality detected	No organ with gross findings
	**500**	No abnormality detected	No organ with gross findings
	**1000**	No abnormality detected	No organ with gross findings
	**2000**	No abnormality detected	No organ with gross findings
**Feale**	**0**	No abnormality detected	No organ with gross findings
	**500**	No abnormality detected	No organ with gross findings
	**1000**	No abnormality detected	No organ with gross findings
	**2000**	No abnormality detected	No organ with gross findings

Rats (*n* = 5 of male and *n* = 5 of female/group) were administrated with different doses of SH003 (0, 500, 1000 and 2000 mg/kg). During 14 days after acute orally administrated, all animals were observed mortality (A), clinical signs and gross finding (B).

Supplementary Table S2: 4-week repeated toxicity of SH003. See Supplementary Table S2 and S3

Supplementary Table S3: 13-week repeated-with a 4-week recovery toxicity of SH003. See Supplementary Table S2 and S3

Supplementary Table S4: The effects of SH003 on the CYP enzyme activity

P450 isozyme	Remaining activities (% of control)						IC^{50}
	SH003 (μg/mL)						
	1	3	10	30	100	300	
**CYP1A2 (Phenacetin-O-deethylation)**	102.94	102.67	98.34	76.25	64.91	52.54	> 300
**CYP2A6 (Coumarin-7-hydroxylation)**	103.31	97.55	89.85	90.08	86.90	85.53	> 300
**CYP2C8 (Paclitaxel-6-hydroxylation)**	97.83	106.06	108.29	90.59	83.50	88.03	> 300
**CYP2C9 (Diclofenac-4-hydroxylation)**	109.38	104.72	105.58	88.79	94.86	96.27	> 300
**CYP2C19 (Mephenytoin-4-hydroxylation)**	103.74	102.12	90.94	81.87	85.48	74.00	> 300
**CYP2D6 (Dextromethorphan-O-demethylation)**	99.52	98.37	91.78	90.37	88.10	81.84	> 300
**CYP3A4 (Midazolam-1-hydroxylation)**	106.61	102.29	95.42	91.98	90.60	84.97	> 300

Human liver microsomes was preincubated with different doses of SH003 (1, 3, 10, 30, 100 and 300 μg/ml) and the substrates (40 μm of phenacetin, 2.5 μm of coumarin, 10 μm of paclitaxel, 10 μm of diclofenac, 160 μm of (±)-mephenytoin, 5 μm of dextromethorphan and 2.5 μm midazolam) were added. Inhibition potential of CYP isozymes (CYP1A2, CYP2A6, CYP2C8, CYP2C9, CYP2C19, CYP2D6 and CYP3A4) by SH003 treatment was measured.
