# Supplementary material for: SH003 suppresses breast cancer growth by accumulating p62 in autolysosomes
Source: Oncotarget. 2016 Aug 19;8(51):88386–400. doi: 10.18632/oncotarget.11393 (PMC5687613; doi:10.18632/oncotarget.11393)
Supplement: Supplementary file 2 [file oncotarget-08-88386-s002.docx]

Table S2. 4-week repeated toxicity of SH003. Rats (*n*=10, 5 rats of each sex) were administrated with different doses of SH003 (0, 500, 1000 and 2000mg/kg) every day. After 4 weeks, all rats were observed mortality (A) clinical signs (B), hematological values (C), serum biochemical values (D) and absolute organ weights (E).

(C) Hematological values

WBC: White blood cell, NEU: Neutrophil, NYM: Lymphocyte, MONO: Monocyte, EOS: Eosinophil, LUC: Large unstained cells, BASO: Basophil, RBC: Red blood cell, HGB: Hemoglobin, HCT: Hematocrit, MCV: Mean corpuscular volume, MCH: Mean corpuscular hemoglobin, MCHC: Mean corpuscular Hb conc, RDW: Red cell distribution width, PLT: Platelet, MPV: Mean platelet volume, Retic: Reticulocyte.

(D) Serum biochemical

AST: Aspartateaminotransferase, ALT: Alanine aminotransferase, T-BIL: Total bilirubin, ALP: Alkaline phosphatase, CPK: Creatine phosphokinase, BUN: Blood urea nitrogen, GLU: Glucose, ALB: Albumin, TP: Total protein, A/G: Albumin/Globulin ratio, CHO: Total cholesterol, CRE: Creatinine, TG: Triglyceride, IP: Inorganic phosphorus, Ca2^+^: Calcium ion, Na^+^: Sodium ion, K^+^: Potassium ion, Cl^-^: Chloride ion.

**Choi et al. Table S2.**

**A**

| **Sex** | **Group (mg/kg)** | **Dead (n)** | **Survive (n)** | **Mortality (%)** | **Days** |
| --- | --- | --- | --- | --- | --- |
| **Male** | **0** | **0** | **5** | **0** | **0~28** |
|  | **500** | **0** | **5** | **0** | **0~28** |
|  | **1000** | **0** | **5** | **0** | **0~28** |
|  | **2000** | **0** | **5** | **0** | **0~28** |
| **Female** | **0** | **0** | **5** | **0** | **0~28** |
|  | **500** | **0** | **5** | **0** | **0~28** |
|  | **1000** | **0** | **5** | **0** | **0~28** |
|  | **2000** | **0** | **5** | **0** | **0~28** |

**B**

| **Sex** | **Group (mg/kg)** | **No. of animals** | **Clinical signs** | **No. of animals affected** | **Days** |
| --- | --- | --- | --- | --- | --- |
| **Male** | **0** | 5 | No abnormality detected | 0 | 0~28 |
|  | **500** | 5 | No abnormality detected | 0 | 0~28 |
|  | **1000** | 5 | No abnormality detected | 0 | 0~28 |
|  | **2000** | 5 | No abnormality detected | 0 | 0~28 |
| **Feale** | **0** | 5 | No abnormality detected | 0 | 0~28 |
|  | **500** | 5 | No abnormality detected | 0 | 0~28 |
|  | **1000** | 5 | No abnormality detected | 0 | 0~28 |
|  | **2000** | 5 | No abnormality detected | 0 | 0~28 |

**C**

| **Sex** | **Dose (mg/kg)** | **0** | **500** | **1000** | **2000** |
| --- | --- | --- | --- | --- | --- |
| **Male** | **WBC (10^3^/μL)** | 10.49±2.66 | 8.53±2.54 | 7.19±1.64 | 9.59±3.15 |
|  | **NEU (%)** | 14.6±7.6 | 12.2±3.9 | 13.5±3.6 | 14.2±2.9 |
|  | **LYM (%)** | 8.23±7.7 | 85.0±3.9 | 82.4±4.1 | 82.9±3.1 |
|  | **MONO (%)** | 1.8±0.5 | 1.4±0.3 | 2.0±0.7 | 1.4±0.1 |
|  | **EOS (%)** | 0.9±0.4 | 0.8±0.2 | 1.6±1.5 | 0.8±0.2 |
|  | **LUC (%)** | 0.4±0.1 | 0.5±0.2 | 0.5±0.2 | 0.6±0.3 |
|  | **BASO (%)** | 0.1±0.0 | 0.1±0.1 | 0.1±0.1 | 0.1±0.1 |
|  | **RBC (10^3^/μL)** | 8.75±0.34 | 8.50±0.30 | 8.42±0.30 | 8.40±0.27 |
|  | **HGB (g/dL)** | 16.7±0.8 | 16.1±036 | 15.9±0.5 | 16.1±0.4 |
|  | **HCT (%)** | 50.9±2.1 | 50.3±1.8 | 50.0±1.6 | 50.0±1.4 |
|  | **MCV (fL)** | 58.2±0.7 | 59.2±1.1 | 59.3±1.5 | 59.6±1.0 |
|  | **MCH (pg)** | 19.1±0.3 | 19.0±0.4 | 18.9±0.7 | 19.2±0.3 |
|  | **MCHC (g/dL)** | 32.8±0.3 | 32.1±0.1 | 31.9±0.4 | 32.2±0.3 |
|  | **RDW (%)** | 11.2±0.3 | 11.5±0.2 | 11.2±0.2 | 11.4±0.2 |
|  | **PLT (10^3^/μL)** | 992±190 | 1183±105 | 949±170 | 945±84 |
|  | **MPV (fL)** | 6.8±0.4 | 6.7±0.2 | 7.4±0.4 | 7.0±0.6 |
|  | **Retic (%)** | 2.24±0.47 | 2.29±0.12 | 2.20±0.23 | 2.09±0.43 |
| **Female** | **WBC (10^3^/μL)** | 6.66±2.23 | 6.78±0.75 | 7.86±2.86 | 7.18±1.98 |
|  | **NEU (%)** | 13.3±4.0 | 8.3±1.7 | 11.0±3.5 | 10.3±7.6 |
|  | **LYM (%)** | 83.8±4.2 | 29.4±1.8 | 86.7±3.5 | 87.0±7.9 |
|  | **MONO (%)** | 1.6±0.3 | 1.0±0.1 | 1.1±0.2 | 1.3±0.5 |
|  | **EOS (%)** | 0.8±0.3 | 0.8±0.2 | 0.7±0.2 | 0.9±0.2 |
|  | **LUC (%)** | 0.6±0.1 | 0.4±0.1 | 0.4±0.2 | 0.4±0.2 |
|  | **BASO (%)** | 13.0±4.0 | 8.3±1.7 | 11.0±3.5 | 10.3±7.6 |
|  | **RBC (10^3^/μL)** | 8.26±0.49 | 8.39±0.53 | 8.23±0.20 | 8.09±0.32 |
|  | **HGB (g/dL)** | 15.6±0.7 | 15.9±0.4 | 15.6±0.6 | 15.4±0.6 |
|  | **HCT (%)** | 47.7±2.1 | 48.5±2.0 | 48.5±1.3 | 47.3±2.8 |
|  | **MCV (fL)** | 57.9±1.2 | 57.9±1.5 | 59.0±0.5 | 57.4±2.5 |
|  | **MCH (pg)** | 18.9±0.5 | 19.0±0.9 | 19.0±0.3 | 19.0±0.5 |
|  | **MCHC (g/dL)** | 32.7±0.5 | 32.8±0.8 | 32.1±0.6 | 32.4±0.6 |
|  | **RDW (%)** | 11.0±0.4 | 11.0±0.3 | 11.4±0.4 | 11.3±0.3 |
|  | **PLT (10^3^/μL)** | 1161±45 | 1.93±70 | 1045±166 | 996±85 |
|  | **MPV (fL)** | 7.3±0.2 | 7.5±0.6 | 8.9±0.6 | 8.8±0.9 |
|  | **Retic (%)** | 2.43±0.43 | 2.27±0.35 | 2.68±0.37 | 2.32±0.27 |

**D**

| **Sex** | **Dose (mg/kg)** | **0** | **500** | **1000** | **2000** |
| --- | --- | --- | --- | --- | --- |
| **Male** | **AST (IU/L)** | 104±17 | 90±6 | 81±8 | 75±7 |
|  | **ALT (IU/L)** | 51±8 | 54±11 | 44±10 | 39±6 |
|  | **T-BIL (mg/dL)** | 0.01±0103 | 0,01±0,01 | 0.01±0.01 | 0.00±0.00 |
|  | **ALP (IU/L)** | 689±131 | 497±99 | 533±126 | 479±75 |
|  | **CPK (IU/L)** | 312±110 | 246±30 | 219±81 | 204±20 |
|  | **BUN (mg/dL)** | 18±3 | 16±1 | 18±2 | 19±2 |
|  | **GLU (mg/dL)** | 128±30 | 143±34 | 123±12 | 146±25 |
|  | **ALB (g/dL)** | 2.6±0.1 | 2.7±0.1 | 2.8±0.1 | 2.8±0.2 |
|  | **TP (g/dL)** | 6.7±0.4 | 6.8±0.3 | 6.8±0.2 | 6.6±0.3 |
|  | **A/G (ratio)** | 0.65±0.05 | 0.67±0.04 | 1.70±0.03 | 0.72±0.03 |
|  | **CHO (mg/dL)** | 77±13 | 88±11 | 91±4 | 94±13 |
|  | **CRE (mg/dL)** | 0.6±0.1 | 0.5±0.0 | 0.6±0.1 | 0.5±0.0 |
|  | **TG (mg/dL)** | 46±17 | 45±18 | 40±13 | 41±16 |
|  | **IP (mg/dL)** | 11.18±1.05 | 10.72±0.47 | 10.95±1.22 | 11.10±0.71 |
|  | **Ca^2+^ (mg/dL)** | 11.3±0.6 | 11.3±0.2 | 11.2±0.2 | 11/4±0.6 |
|  | **Na^+^ (mmol/L)** | 145±2 | 145±1 | 143±1 | 143±1 |
|  | **K^+^ (mmol/L)** | 6.9±1.1 | 6.2±0.3 | 7.0±2.1 | 6.4±0.8 |
|  | **CI^-^ (mmol/L)** | 104±1 | 105±2 | 104±1 | 106±1 |
| **Female** | **AST (IU/L)** | 8±5 | 105±8 | 96±12 | 91±11 |
|  | **ALT (IU/L)** | 37±6 | 50±7 | 51±17 | 49±9 |
|  | **T-BIL (mg/dL)** | 0.04±0.02 | 0.06±0.03 | 0.04±0.03 | 0.04±0.01 |
|  | **ALP (IU/L)** | 277±79 | 269±7 | 298±37 | 278±34 |
|  | **CPK (IU/L)** | 191±48 | 196±7 | 208±57 | 151±31 |
|  | **BUN (mg/dL)** | 21±6 | 17±3 | 16±3 | 15±3 |
|  | **GLU (mg/dL)** | 141±24 | 125±14 | 123±25 | 146±25 |
|  | **ALB (g/dL)** | 3.2±0.3 | 3.0±0.1 | 3.1±0.1 | 3.3±0.4 |
|  | **TP (g/dL)** | 7.3±0.5 | 6.9±0.3 | 7.1±0.2 | 7.4±0.6 |
|  | **A/G (ratio)** | 0.78±0.06 | 0.79±0.03 | 0.76±0.03 | 0.82±0.05 |
|  | **CHO (mg/dL)** | 99±22 | 104±2 | 107±26 | 113±9 |
|  | **CRE (mg/dL)** | 0.6±.0.1 | 0.5±0.1 | 0.6±0.0 | 0.6±0.1 |
|  | **TG (mg/dL)** | 37±18 | 41±19 | 31±15 | 34±20 |
|  | **IP (mg/dL)** | 9.02±1.26 | 8.92±1.39 | 8.43±0.37 | 8.72±1.25 |
|  | **Ca^2+^ (mg/dL)** | 10.9±0.4 | 10.5±0.7 | 10.4±0.3 | 11.0±0.9 |
|  | **Na^+^ (mmol/L)** | 145±2 | 145±3 | 145±2 | 143±3 |
|  | **K^+^ (mmol/L)** | 6.0±1.3 | 5.7±1.6 | 5.1±0.8 | 6.0±1.6 |
|  | **CI^-^ (mmol/L)** | 105±1 | 106±1 | 105±1 | 106±2 |

**E**

| **Sex** | **Dose (mg/kg)** | **0** | **500** | **1000** | **2000** |
| --- | --- | --- | --- | --- | --- |
| **Male** | **Brain** | 0.4636±0.0339 | 0.4587±0.019 | 0.4447±0.0178 | 0.4541±0.0287 |
|  | **Liver** | 3.0993±0.1043 | 3.3576±0.2114 | 3.501±0.0944 | 3.6341±0.1906 |
|  | **Spleen** | 0.1871±0.0335 | 0.1692±0.0172 | 0.1751±0.0212 | 0.1862±0.0307 |
|  | **Kidney-L** | 0.37±0.0306 | 0.3755±0.0334 | 0.37850±0.0339 | 0.379±0.0224 |
|  | **Kidney-R** | 0.365±0.0304 | 0.3803±0.0307 | 0.3772±0.0335 | 0.3843±0.0199 |
|  | **Heart** | 0.3109±0.0238 | 0.3336±0.0202 | 0.3274±0.0186 | 0.3195±0.0167 |
|  | **Lung** | 0.3603±0.0398 | 0.36±0.0143 | 0.3384±0.0163 | 0.3587±0.0116 |
| **Female** | **Brain** | 0.7142±0.0472 | 0.7213±0.0334 | 0.7378±0.0274 | 0.7263±0.0104 |
|  | **Liver** | 2.8985±0.500 | 3.1969±0.1020 | 3.4797±0.3313 | 3.7687±0.2634 |
|  | **Spleen** | 0.2138±0.0266 | 0.2044±0.0205 | 0.2118±0.0202 | 0.2107±0.0278 |
|  | **Kidney-L** | 0.3381±0.0375 | 0.3421±0.0226 | 0.3340±0.0176 | 0.3512±0.0278 |
|  | **Kidney-R** | 0.3489±0.0359 | 0.3503±0.0330 | 0.3356±0.0308 | 0.3696±0.0349 |
|  | **Heart** | 0.3340±0.0196 | 0.3432±0.0236 | 0.3398±0.017 | 0.3575±0.0194 |
|  | **Lung** | 0.4557±0.0261 | 0.4623±0.0221 | 0.4664±0.0156 | 0.4694±0.0428 |

Table S3. 13-week repeated-with a 4-week recovery toxicity of SH003. Male and female rates were divided in to 4 groups: the control (*n*=15), 62.5mg/kg of SH003 (*n*=10), 1250mg/kg of SH003 (*n*=10) and 2500mg/kg of SH003 (*n*=15). Animals were orally administrated with distilled water or SH003 daily. After 13 weeks, five rats both male and female from the control and 2500mg/kg of SH003 group were selected and recovered for 4 weeks. All rates were observed mortality (A), clinical signs (B), hematological values (C), serum biochemical values (D) and absolute organ weights (E).

(C) Hematological values

WBC: White blood cell, NEU: Neutrophil, NYM: Lymphocyte, MONO: Monocyte, EOS: Eosinophil, LUC: Large unstained cells, BASO: Basophil, RBC: Red blood cell, HGB: Hemoglobin, HCT: Hematocrit, MCV: Mean corpuscular volume, MCH: Mean corpuscular hemoglobin, MCHC: Mean corpuscular Hb conc, RDW: Red cell distribution width, PLT: Platelet, MPV: Mean platelet volume, Retic: Reticulocyte.

(D) Serum biochemical

AST: Aspartateaminotransferase, ALT: Alanine aminotransferase, T-BIL: Total bilirubin, ALP: Alkaline phosphatase, CPK: Creatine phosphokinase, BUN: Blood urea nitrogen, GLU: Glucose, ALB: Albumin, TP: Total protein, A/G: Albumin/Globulin ratio, CHO: Total cholesterol, CRE: Creatinine, TG: Triglyceride, IP: Inorganic phosphorus, Ca2^+^: Calcium ion, Na^+^: Sodium ion, K^+^: Potassium ion, Cl^-^: Chloride ion.

**Choi et al. Table S3.**

**A**

| **Sex** | **Group (mg/kg)** | **Dead (n)** | **Survive (n)** | **Mortality (%)** | **Days** |
| --- | --- | --- | --- | --- | --- |
| **Male** | **0** | 0 | 10 | 0 | 0~90 |
|  | **625** | 0 | 10 | 0 | 0~90 |
|  | **1250** | 0 | 10 | 0 | 0~90 |
|  | **2500** | 0 | 10 | 0 | 0~90 |
|  | **Recovery-0** | 0 | 5 | 0 | 0~118 |
|  | **Recovery-2500** | 0 | 5 | 0 | 0~118 |
| **Female** | **0** | 0 | 10 | 0 | 0~90 |
|  | **625** | 0 | 10 | 0 | 0~90 |
|  | **1250** | 0 | 10 | 0 | 0~90 |
|  | **2500** | 0 | 10 | 0 | 0~90 |
|  | **Recovery-0** | 0 | 5 | 0 | 0~118 |
|  | **Recovery-2500** | 0 | 5 | 0 | 0~118 |

**B**

| **Sex** | **Group (mg/kg)** | **No. of animals** | **Clinical signs** | **No. of animals affected** | **Days** |
| --- | --- | --- | --- | --- | --- |
| **Male** | **0** | 10 | No abnormality detected | 0 | 0~90 |
|  | **625** | 10 | No abnormality detected | 0 | 0~90 |
|  | **1250** | 10 | No abnormality detected | 0 | 0~90 |
|  | **2500** | 10 | No abnormality detected | 0 | 0~90 |
|  | **Recovery-0** | 5 | No abnormality detected | 0 | 0~118 |
|  | **Recovery-2500** | 5 | No abnormality detected | 0 | 0~118 |
| **Female** | **0** | 10 | No abnormality detected | 0 | 0~90 |
|  | **625** | 10 | No abnormality detected | 0 | 0~90 |
|  | **1250** | 10 | No abnormality detected | 0 | 0~90 |
|  | **2500** | 10 | No abnormality detected | 0 | 0~90 |
|  | **Recovery-0** | 5 | No abnormality detected | 0 | 0~118 |
|  | **Recovery-2500** | 5 | No abnormality detected | 0 | 0~118 |

**C**

| **Sex** | **Dose (mg/kg)** | **0** | **625** | **1250** | **2500** | **Recovery-0** | **Recovery-2500** |
| --- | --- | --- | --- | --- | --- | --- | --- |
| **Male** | **WBC (10^3^/μL)** | 10.12±2.39 | 8.28±1.64 | 8.73±3.07 | 9.18±1.50 | 6.91±0.85 | 5.35±1.55 |
|  | **NEU (%)** | 12.3±2.0 | 14.8±4.4 | 13.03.5 | 14.4±3.3 | 14.4±3.4 | 17.4±6.9 |
|  | **LYM (%)** | 83.7±2.5 | 81.1±4.7 | 83.0±3.7 | 81.8±3.6 | 82.8±3.7 | 79.3±7.4 |
|  | **MONO (%)** | 2.0±0.4 | 2.1±0.5 | 2.2±0.6 | 2.0±0.7 | 1.4±0.4 | 1.7±0.5 |
|  | **EOS (%)** | 0.9±0.2 | 1.0±0.4 | 0.6±0.2 | 0.7±0.4 | 0.8±0.2 | 0.9±0.3 |
|  | **LUC (%)** | 1.0±0.5 | 1.0±0.5 | 1.2±0.2 | 1.0±0.2 | 0.6±0.2 | 0.6±0.3 |
|  | **BASO (%)** | 0.1±0.1 | 0.1±0.0 | 0.1±0.1 | 0.1±0.1 | 0.1±0.1 | 0.1±0.0 |
|  | **RBC (10^3^/μL)** | 9.15±0.36 | 8.84±0.55 | 9.02±0.39 | 8.81±0.26 | 8.94±0.75 | 9.16±0.34 |
|  | **HGB (g/dL)** | 15.9±0.4 | 15.3±0.7 | 15.3±0.5 | 14.8±0.5 | 15.6±1.3 | 15.5±0.7 |
|  | **HCT (%)** | 48.2±1.8 | 46.6±2.7 | 46.3±1.8 | 45.2±1.4 | 48.7±4.1 | 49.0±2.4 |
|  | **MCV (fL)** | 52.8±1.0 | 52.7±1.3 | 51.4±1.6 | 51.3±1.4 | 54.4±1.0 | 53.5±1.8 |
|  | **MCH (pg)** | 17.4±0.4 | 17.3±0.6 | 17.0±0.6 | 16.8±0.4 | 17.5±0.2 | 16.9±0.3 |
|  | **MCHC (g/dL)** | 33.0±0.5 | 32.8±0.5 | 33.1±0.5 | 32.8±0.4 | 32.1±0.4 | 31.6±0.8 |
|  | **RDW (%)** | 12.4±0.5 | 12.5±0.5 | 12.6±0.6 | 13.2±0.4 | 12.8±0.5 | 13.5±0.9 |
|  | **PLT (10^3^/μL)** | 1206±143 | 1191±180 | 1128±156 | 1184±144 | 1118±69 | 1180±221 |
|  | **MPV (fL)** | 6.9±0.4 | 6.6±0.6 | 6.8±0.4 | 7.0±0.3 | 9.6±0.2 | 9.5±0.6 |
|  | **Retic (%)** | 19.2±0.24 | 1.82±0.21 | 1.96±0.32 | 1.78±0.32 | 1.94±0.25 | 1.99±0.47 |
| **Female** | **WBC (10^3^/μL)** | 6.89±2.33 | 4.86±1.39 | 5.99±1.55 | 6.98±1.55 | 6.13±1.46 | 4.05±1.31 |
|  | **NEU (%)** | 11.8±3.8 | 13.4±6.4 | 9.6±2.5 | 10.5±3.2 | 11.4±4.1 | 15.3±5.9 |
|  | **LYM (%)** | 83.8±4.2 | 82.9±6.7 | 86.7±2.8 | 85.6±3.6 | 84.8±4.4 | 80.2±7.2 |
|  | **MONO (%)** | 2.4±0.7 | 1.7±0.3 | 1.6±0.6 | 1.8±0.6 | 2.0±0.3 | 2.6±1.3 |
|  | **EOS (%)** | 1.1±0.5 | 1.2±0.6 | 1.0±0.3 | 1.0±0.4 | 0.9±0.6 | 0.9±0.1 |
|  | **LUC (%)** | 0.8±0.2 | 0.8±0.2 | 1.0±0.5 | 1.0±0.3 | 0.7±0.2 | 0.9±0.1 |
|  | **BASO (%)** | 0.1±0.1 | 0.1±0.1 | 0.1±0.1 | 0.2±0.1 | 0.1±0.0 | 0.1±0.0 |
|  | **RBC (10^3^/μL)** | 8.23±0.27 | 8.15±0.51 | 8.12±0.49 | 8.06±0.32 | 8.41±0.59 | 8.13±0.26 |
|  | **HGB (g/dL)** | 15.5±0.5 | 15.1±0.7 | 14.6±0.7 | 14.7±0.6 | 15.7±0.6 | 15.1±0.9 |
|  | **HCT (%)** | 45.1±1.4 | 44.5±2.5 | 43.9±2.3 | 43.9±1.8 | 47.6±2.3 | 45.4±2.6 |
|  | **MCV (fL)** | 54.8±1.0 | 54.6±2.0 | 54.1±1.2 | 54.5±2.1 | 56.6±1.5 | 55.8±2.2 |
|  | **MCH (pg)** | 18.9±0.5 | 18.5±0.8 | 18.1±0.6 | 18.3±0.6 | 18.7±0.6 | 18.5±0.9 |
|  | **MCHC (g/dL)** | 34.4±0.5 | 33.9±0.5 | 33.4±0.6 | 33.6±0.5 | 33.1±0.6 | 33.2±0.6 |
|  | **RDW (%)** | 11.3±0.3 | 11.6±0.4 | 12.2±2.1 | 11.7±0.4 | 11.9±0.4 | 12.3±0.3 |
|  | **PLT (10^3^/μL)** | 1027±135 | 1022±162 | 1088±169 | 1167±164 | 924±85 | 1174±121 |
|  | **MPV (fL)** | 6.6±1.2 | 7.0±1.1 | 7.3±0.8 | 7.3±0.8 | 8.4±0.3 | 8.0±0.3 |
|  | **Retic (%)** | 1.93±0.35 | 1.84±0.34 | 1.74±0.41 | 2.01±0.36 | 2.29±0.32 | 2.01±0.11 |

**D**

| **Sex** | **Dose (mg/kg)** | **0** | **625** | **1250** | **2500** | **Recovery-0** | **Recovery-2500** |
| --- | --- | --- | --- | --- | --- | --- | --- |
| **Male** | **ALT (IU/L)** | 46±9 | 38±10 | 46±7 | 38±7 | 44±28 | 30±3 |
|  | **AST (IU/L)** | 100±18 | 75±12 | 86±14 | 76±12 | 114±53 | 80±9 |
|  | **ALP (IU/L)** | 317±50 | 254±71 | 279±74 | 236±31 | 241±38 | 196±20 |
|  | **T-BIL (mg/dL)** | 0.03±0.04 | 0.02±0.01 | 0.01±0.01 | 0.02±0.01 | 0.02±0.01 | 0.01±0.01 |
|  | **BUN (mg/dL)** | 22±3 | 18±2 | 23±5 | 22±3 | 16±3 | 18±1 |
|  | **CRE (mg/dL)** | 0.7±0.1 | 0.7±0.1 | 0.7±0.1 | 0.7±0.1 | 0.6±0.1 | 0.6±0.1 |
|  | **CHO (mg/dL)** | 90±15 | 96±25 | 99±24 | 116±26 | 80±33 | 80±11 |
|  | **TP (g/dL)** | 7.3±0.4 | 7.1±0.6 | 7.5±0.3 | 7.5±0.4 | 6.8±0.5 | 6.8±0.4 |
|  | **CPK (IU/L)** | 375±217 | 239±75 | 332±203 | 226±94 | 371±122 | 272±117 |
|  | **TG (mg/dL)** | 69±23 | 113±50 | 66±33 | 65±20 | 49±29 | 52±11 |
|  | **ALB (g/dL)** | 2.8±0.1 | 2.9±0.2 | 3.0±0.2 | 3.2±0.3 | 2.7±0.3 | 2.7±0.1 |
|  | **GLU (mg/dL)** | 123±23 | 131±34 | 113±19 | 115±13 | 126±15 | 143±42 |
|  | **A/G (ratio)** | 0.64±0.06 | 0.67±0.04 | 0.68±0.03 | 0.72±0.06 | 0.61±0.02 | 0.61±0.02 |
|  | **IP (mg/dL)** | 10.32±1.36 | 10.05±1.02 | 9.66±0.95 | 9.61±0.63 | 7.38±0.43 | 7.92±1.26 |
|  | **Ca^2+^ (mg/dL)** | 11.2±0.4 | 11.0±0.5 | 10.8±0.4 | 10.8±0.4 | 9.6±0.5 | 10.0±0.5 |
|  | **Na^+^ (mmol/L)** | 147±2 | 146±3 | 146±2 | 145±2 | 143±1 | 145±2 |
|  | **K^+^ (mmol/L)** | 7.2±2.0 | 6.9±1.3 | 6.3±1.3 | 6.6±0.9 | 5.0±1.1 | 5.2±1.3 |
|  | **CI^-^ (mmol/L)** | 105±3 | 105±1 | 105±2 | 106±2 | 102±2 | 103±1 |
| **Female** | **ALT (IU/L)** | 52±22 | 46±9 | 39±7 | 35±8 | 61±35 | 27±5 |
|  | **AST (IU/L)** | 116±68 | 79±13 | 76±14 | 68±10 | 134±80 | 72±9 |
|  | **ALP (IU/L)** | 158±32 | 171±51 | 161±77 | 162±39 | 119±14 | 102±8 |
|  | **T-BIL (mg/dL)** | 0.07±0.03 | 0.04±0.01 | 0.03±0.02 | 0.04±0.01 | 0.07±0.01 | 0.01±0.01 |
|  | **BUN (mg/dL)** | 22±5 | 23±4 | 18±3 | 26±6 | 20±5 | 22±4 |
|  | **CRE (mg/dL)** | 0.7±0.1 | 0.7±0.1 | 0.70.1 | 0.70.1 | 0.8±0.1 | 0.7±0.1 |
|  | **CHO (mg/dL)** | 99±19 | 120±15 | 141±29 | 146±24 | 94±10 | 87±22 |
|  | **TP (g/dL)** | 7.4±0.5 | 7.3±0.3 | 7.70.4 | 7.80.5 | 7.40.8 | 6.9±0.4 |
|  | **CPK (IU/L)** | 220±63 | 229±94 | 225±113 | 165±39 | 235±68 | 140±32 |
|  | **TG (mg/dL)** | 53±23 | 52±15 | 43±15 | 62±29 | 51±18 | 33±21 |
|  | **ALB (g/dL)** | 3.2±0.3 | 3.2±0.2 | 3.50.2 | 3.3±0.3 | 3.2±0.4 | 3.0±0.3 |
|  | **GLU (mg/dL)** | 133±24 | 127±22 | 132±13 | 134±18 | 154±35 | 97±21 |
|  | **A/G (ratio)** | 0.76±0.05 | 0.79±0.04 | 0.820.04 | 0.85±0.06 | 0.56±0.03 | 0.57±0.03 |
|  | **IP (mg/dL)** | 8.92±0.91 | 9.07±0.81 | 9.151.48 | 9.16±0.88 | 7.74±1.23 | 7.68±1.02 |
|  | **Ca^2+^ (mg/dL)** | 10.9±0.4 | 10.6±0.4 | 10.9±0.5 | 11.1±0.6 | 10.8±1.1 | 10.0±0.6 |
|  | **Na^+^ (mmol/L)** | 145±1 | 144±1 | 145±2 | 145±2 | 145±2 | 144±1 |
|  | **K^+^ (mmol/L)** | 6.4±1.3 | 6.3±1.2 | 6.31.3 | 7.6±1.0 | 5.3±1.2 | 6.2±1.1 |
|  | **CI^-^ (mmol/L)** | 105±2 | 106±1 | 107±3 | 107±2 | 103±1 | 105±2 |

**E**

| **Sex** | **Dose (mg/kg)** | **0** | **625** | **1250** | **2500** | **Recovery-0** | **Recovery-2500** |
| --- | --- | --- | --- | --- | --- | --- | --- |
| **Male** | **Pituitary** | 0.0023±0.0004 | 0.0022±0.0004 | 0.0024±0.0002 | 0.0023±0.0004 | 0.0026±0.0004 | 0.0026±0.0004 |
|  | **Adrenal-L** | 0.0060±0.0009 | 0.0055±0.0011 | 0.0057±0.0008 | 0.0054±0.0010 | 0.0054±0.0010 | 0.0056±0.0008 |
|  | **Adrenal-R** | 0.0056±0.0008 | 0.0054±0.0007 | 0.0056±0.0009 | 0.0055±0.0012 | 0.0052±0.0009 | 0.0058±0.0009 |
|  | **Brain** | 0.3625±0.0290 | 0.3710±0.0249 | 0.3844±0.0294 | 0.3789±0.0252 | 0.3900±0.0508 | 0.3852±0.0282 |
|  | **Liver** | 2.7290±0.1898 | 3.0932±0.2278 | 3.3025±0.2647 | 3.8447±0.1563 | 2.7474±0.1459 | 2.8623±0.1834 |
|  | **Spleen** | 0.1583±0.0202 | 0.1633±0.0224 | 0.1703±0.0253 | 0.1543±0.0152 | 0.1607±0.0143 | 0.1384±0.0214 |
|  | **Kidney-L** | 0.3152±0.0137 | 0.3387±0.0402 | 0.3450±0.0211 | 0.3885±0.0321 | 0.2920±0.0183 | 0.3148±0.0173 |
|  | **Kidney-R** | 0.3199±0.0177 | 0.3398±0.0414 | 0.3512±0.0128 | 0.3813±0.0239 | 0.2952±0.0141 | 0.3230±0.0181 |
|  | **Heart** | 0.2743±0.0157 | 0.2924±0.0178 | 0.2932±0.0192 | 0.3036±0.0140 | 0.2900±0.0182 | 0.2976±0.0068 |
|  | **Thymus** | 0.0566±0.0059 | 0.0431±0.0089 | 0.0590±0.0136 | 0.0446±0.0127 | 0.0396±0.0094 | 0.0415±0.0095 |
|  | **Lung** | 0.3063±0.0222 | 0.3188±0.0260 | 0.3298±0.0152 | 0.3388±0.0233 | 0.3228±0.0400 | 0.3353±0.0200 |
|  | **Prostate** | 0.1234±0.0196 | 0.1222±0.0240 | 0.1299±0.0360 | 0.1343±0.0212 | 0.1932±0.1911 | 0.1252±0.0191 |
|  | **Salivary gland-L** | 0.0673±0.0072 | 0.074±0.0076 | 0.0715±0.0086 | 0.0727±0.0076 | 0.0693±0.0091 | 0.0725±0.0095 |
|  | **Salivary gland-R** | 0.0642±0.0085 | 0.0719±0.0091 | 0.0769±0.0208 | 0.0733±0.0083 | 0.0670±0.0121 | 0.0708±0.0097 |
|  | **Testis-L** | 0.2936±0.0361 | 0.3076±0.0379 | 0.3368±0.0340 | 0.3306±0.0349 | 0.2942±0.0512 | 0.3340±0.0193 |
|  | **Testis-R** | 0.2929±0.0369 | 0.3031±0.0366 | 0.3359±0.0308 | 0.3334±0.0301 | 0.2926±0.0513 | 0.3357±0.0258 |
|  | **Epididymis-L** | 0.1298±0.0097 | 0.1288±0.0154 | 0.1384±0.0066 | 0.1304±0.0133 | 0.1405±0.0279 | 0.1333±0.0046 |
|  | **Epididymis-R** | 0.1311±0.0090 | 0.1308±0.0153 | 0.1416±0.0140 | 0.1303±0.0125 | 0.1440±0.0306 | 0.1431±0.0149 |
| **Female** | **Pituitary** | 0.0053±0.0005 | 0.0057±0.0010 | 0.0057±0.0016 | 0.0058±0.0012 | 0.0052±0.0011 | 0.0023±0.0008 |
|  | **Adrenal-L** | 0.0121±0.0016 | 0.0114±0.0011 | 0.0129±0.0021 | 0.0126±0.0020 | 0.0116±0.0015 | 0.0110±0.0014 |
|  | **Adrenal-R** | 0.0122±0.0018 | 0.0110±0.0023 | 0.0122±0.0019 | 0.0126±0.0016 | 0.0117±0.0014 | 0.0104±0.018 |
|  | **Ovary-L** | 0.0151±0.0029 | 0.0150±0.0026 | 0.0161±0.0045 | 0.0181±0.0039 | 0.0141±0.0008 | 0.0172±0.0015 |
|  | **Ovary-R** | 0.0147±0.0025 | 0.0145±0.0032 | 0.0154±0.0035 | 0.0180±0.0047 | 0.0115±0.0033 | 0.0177±0.0015 |
|  | **Brain** | 0.6416±0.0659 | 0.6621±0.0339 | 0.6692±0.0414 | 0.6800±0.0604 | 0.6282±0.0300 | 0.6987±0.0678 |
|  | **Liver** | 2.6533±0.2416 | 3.1163±0.1899 | 3.5104±0.4609 | 3.9736±0.2288 | 2.5044±0.1198 | 2.7653±0.2154 |
|  | **Spleen** | 0.1783±0.0176 | 0.1805±0.0177 | 0.1999±0.0309 | 0.1951±0.0187 | 0.1697±0.0202 | 0.1873±0.0259 |
|  | **Kidney-L** | 0.3223±0.0235 | 0.3330±0.0179 | 0.3531±0.0273 | 0.3662±0.0219 | 0.3087±0.0230 | 0.3439±0.0139 |
|  | **Kidney-R** | 0.3262±0.0229 | 0.3340±0.0232 | 0.3564±0.0366 | 0.3852±0.0255 | 0.3109±0.0225 | 0.3481±0.0204 |
|  | **Heart** | 0.3010±0.0257 | 0.3292±0.0167 | 0.3550±0.0437 | 0.3507±0.0222 | 0.3010±0.0232 | 0.3492±0.0103 |
|  | **Thymus** | 0.0878±0.0202 | 0.0860±0.0174 | 0.0996±0.0196 | 0.0956±0.0233 | 0.0654±0.0040 | 0.0813±0.0163 |
|  | **Lung** | 0.4376±0.0379 | 0.4448±0.0230 | 0.4779±0.0466 | 0.4475±0.0291 | 0.4190±0.0210 | 0.4787±0.575 |
|  | **Uterus** | 0.2538±0.1677 | 0.2221±0.0784 | 0.2501±0.1384 | 0.3439±0.2524 | 0.3852±0.1584 | 0.2939±0.1463 |
|  | **Salivary gland-L** | 0.0757±0.0095 | 0.0837±0.0085 | 0.0871±0.0105 | 0.0855±0.0083 | 0.0723±0.0018 | 0.0895±0.0154 |
|  | **Salivary gland-R** | 0.0774±0.0089 | 0.0822±0.0103 | 0.0861±0.0086 | 0.0857±0.0062 | 0.0744±0.0053 | 0.0897±0.0128 |
